# Supplementary material for: Chirality coupling in topological magnetic textures with multiple magnetochiral parameters
Source: Nat Commun. 2023 Mar 17;14:1491. doi: 10.1038/s41467-023-37081-z (PMC10023801; doi:10.1038/s41467-023-37081-z)
Supplement: Supplementary file 1 — supplementary information [file 41467_2023_37081_MOESM1_ESM.pdf]

Chirality coupling in topological magnetic textures with multiple magnetochiral parameters

Oleksii M. Volkov,[1, *] Daniel Wolf,[2, †] Oleksandr V. Pylypovskyi,[1, 3] Attila Kákay,[1]
Denis D. Sheka,[4] Bernd Büchner,[2, 5, 6] Jürgen Fassbender,[1] Axel Lubk,[2, 5, 6] and Denys Makarov[1, ‡]
[1] *Helmholtz-Zentrum Dresden-Rossendorf e.V., Institute of Ion Beam Physics and Materials Research, Bautzner Landstr. 400, 01328 Dresden, Germany*
[2] *Institute for Solid State Research, IFW Dresden, 01069 Dresden, Germany*
[3] *Kyiv Academic University, 03142 Kyiv, Ukraine*
[4] *Taras Shevchenko National University of Kyiv, 01601 Kyiv, Ukraine*
[5] *Institute of Solid State and Materials Physics, TU Dresden, 01069 Dresden, Germany*
[6] *Würzburg-Dresden Cluster of Excellence ct.qmat, Germany*
(Dated: March 13, 2023)

The supplementary information provides details on analytics and numerical calculations as well as further analysis of the experimental data.

Supplementary Note 1. Method to determine geometry of the vortex string

A magnetic vortex distribution in a soft ferromagnetic nanodisk contains the in-plane component with the magnetization flux circulation counter-clockwise ($C = +1$) or clockwise ($C = -1$) and out-of-plane component oriented either up ($P = +1$) or down ($P = -1$) [1–3]. Here, C is the circulation and P is the polarity of the magnetic vortex.

There are several methods for the determination of the position of the vortex string, which we illustrate in Supplementary Fig. 1 for a nanodisk with flat top and bottom surfaces as well as a nanodisk with a flat bottom surface and a top surface possessing an off-centered Gaussian bump

$$z = t \exp\left[-\frac{(x-x_0)^2 + y^2}{2b^2}\right], \qquad \text{(3, main text)}$$

where t is the bump height, b is its width and x_0 is the bump shift from the center of the nanodisk. A widely used method for flat disks is based on the analysis of the intersection between $m_x = 0$ and $m_y = 0$ isosurfaces, which defines the spatial location of magnetization with $m_z = 1$ [4]. Although this method is accurate for the case of flat symmetric nanodisks with straight vortex line along $\hat{\boldsymbol{z}}$ direction (Fig. 1a), it fails if the vortex string is sufficiently bent to approach the horizontal plane. Thus, in the case of asymmetric disks with an off-centered Gaussian bump, this approach may give different location of the locus of the vortex string compared to another method, which is based on the computation of the flux components of the topological charge [5–8] (Supplementary Fig. 1b):

$$\begin{aligned}
\Omega_x =& \frac{1}{4\pi}\left[m_x\left(\partial_y m_y \partial_z m_z - \partial_z m_y \partial_y m_z\right) + m_y\left(\partial_y m_z \partial_z m_x - \partial_z m_z \partial_y m_x\right) + m_z\left(\partial_y m_x \partial_z m_y - \partial_z m_x \partial_y m_y\right)\right],\\
\Omega_y =& \frac{1}{4\pi}\left[m_x\left(\partial_z m_y \partial_x m_z - \partial_x m_y \partial_z m_z\right) + m_y\left(\partial_z m_z \partial_x m_x - \partial_x m_z \partial_z m_x\right) + m_z\left(\partial_z m_x \partial_x m_y - \partial_x m_x \partial_z m_y\right)\right],\\
\Omega_z =& \frac{1}{4\pi}\left[m_x\left(\partial_x m_y \partial_y m_z - \partial_y m_y \partial_x m_z\right) + m_y\left(\partial_x m_z \partial_y m_x - \partial_y m_z \partial_x m_x\right) + m_z\left(\partial_x m_x \partial_y m_y - \partial_y m_x \partial_x m_y\right)\right].
\end{aligned} \qquad \text{(S1)}$$

The flux density of the topological charge $\Omega = \sqrt{\Omega_x^2 + \Omega_y^2 + \Omega_z^2}$ reaches its maximum at the topological texture. In the following, we will use the normalized topological charge flux density $\widetilde{\Omega} = \Omega/\Omega_{\max}$, where $\Omega_{\max} = \max|\Omega|$.

In our micromagnetic simulations of the equilibrium vortex states of different magnetochiralities $\widetilde{C} = PC$, we mainly utilize the successor of the GPU accelerated TetraMag and Magpar simulation packages that are based on the finite element method (FEM) techniques with the spatial discretization of geometry by tetrahedral elements, whose lateral size is varied between 1 and 3 nm depending on the sample size. Thus, the numerical value of $\widetilde{\Omega}$ is determined as the average in the center of mass for each tetrahedron, which (in the case of a single mesh) may lead to a rather

---

* o.volkov@hzdr.de
† d.wolf@ifw-dresden.de
‡ d.makarov@hzdr.de

rough spatial localization of the vortex string. The localization accuracy could be improved by using several different meshes with various spatial arrangements of tetrahedron elements along the vortex string. We achieve this goal by means of a slight variation of the average tetrahedron size in each mesh and collecting all spatial distributions of $\widetilde{\Omega}$ into one dataset possessing effectively denser mesh. This gives a substantial improvement of the spatial localization of the vortex string.

An efficient method to determine the locus of the topologically non-trivial texture (the Bloch line in our case) relies on the determining of the maximum of the first moment (center of mass) of the topological charge within the given plane [5, 8–10]. This approach straightforwardly extends to operations with $\widetilde{\Omega}$ and is convenient for numerical processing of the topological magnetic textures in 3D. The algorithm to find the shape of the vortex string consists of the following steps:

1. We select a slice of the geometry being half of the tetrahedron size (1.5 nm), which is parallel to the bottom sample's surface and contains it.

2. The first moment $\boldsymbol{\gamma}_i$ of i-th slice with $i = 1$ at this step is determined as follows:

$$\boldsymbol{\gamma}_i = \frac{\sum_{k=1}^{p} \boldsymbol{r}_k \widetilde{\Omega}^k}{\sum_{k=1}^{p} \widetilde{\Omega}^k}, \tag{S2}$$

   where tetrahedrons are enumerated by the index k, the radius-vector $\boldsymbol{r}_k$ determines the centre of mass of k-th tetrahedron, and the total number of tetrahedrons is p. In this way, we analyse the list of p tetrahedrons satisfying the criterion $|\widetilde{\Omega}| \geq \widetilde{\Omega}_{\text{tr}}$ within the i-th slice with $\widetilde{\Omega}_{\text{tr}}$ being the threshold value of the normalized topological charge density.

3. Steps 1 and 2 are repeated for the second slice just above the first one to obtain $\boldsymbol{\gamma}_2$. The direction of the vortex string is determined by the vector $\boldsymbol{n}_{\text{vor}}^{i=1} = \boldsymbol{\gamma}_2 - \boldsymbol{\gamma}_1$.

4. The normal to the $(i+1)$-th slice ($i \geq 2$) with thickness of about 1.5 nm is selected to be along $\boldsymbol{n}_{\text{vor}}^{i-1}$ determined at the previous step. The point lying at the vortex string $\boldsymbol{\gamma}_{i+1}$ is determined by Eq. (S2).

5. The step 4 is repeated until the sample's boundary is reached.

The vortex string is perpendicular to the bottom surface of the sample due to the exchange-driven boundary conditions. This allows to define the first slices to be parallel to the bottom sample surface. The slice thickness is selected as the balance between the number of points determining the geometry of the vortex string (thicker slices reduce the spatial discretization) and the requirement to have enough tetrahedrons whose centers of mass are in the vicinity of the vortex string and inside the given slice. For a bulk (or large enough slice), the threshold $\widetilde{\Omega}_{\text{tr}}$ value can be selected as 0 to use all available data, which makes the result smoother. However, for asymmetric samples or bent topological textures, the geometrical asymmetry of the slice leads to the bias. To avoid this, we use $\widetilde{\Omega}_{\text{tr}} \sim 0.05 \div 0.1$ to keep roughly the same number of points to determine the first moment of $\widetilde{\Omega}$ in all slices and keep accuracy of calculations the same along $\boldsymbol{\gamma}$. Comparison of the vortex strings determined by the method of isosurfaces and the topological charge flux is shown in Supplementary Fig. 1. In a flat sample (Supplementary Fig. 1a,c,e) both methods give the same results. However, in the asymmetric sample, the origin of magnetization circulated at the bump side lies apart from the point of magnetization along $\hat{\boldsymbol{z}}$ (Fig. 1b,d,f). In this case, both methods give the same results near the bottom sample's surface only (Supplementary Fig. 1f). We note that in the absence of extreme bends of the vortex strings, the method of isosurfaces still can be used as an estimation of the string shape. Namely, the vortex string determined by the proposed method goes directly to the point of magnetization curling at the top surface, while the red one is somewhere around, which confirms that the method of topological flux density provides a more reliable result.

The vortex string can be treated in the following analysis as a space curve with the respective local orthogonal TNB reference frame formed by the tangential (T), $\boldsymbol{e}_{\text{T}}$, normal (N), $\boldsymbol{e}_{\text{N}}$, and binormal (B), $\boldsymbol{e}_{\text{B}}$, unit vectors. The discrete set of points $\boldsymbol{\gamma} = \{\boldsymbol{\gamma}_i\}$ with $i = \overline{1, N}$ and N gives the tangential unit vector [11]

$$\boldsymbol{e}_{\text{T}}^i = \frac{\boldsymbol{\gamma}_{i+1} - \boldsymbol{\gamma}_i}{\|\boldsymbol{\gamma}_{i+1} - \boldsymbol{\gamma}_i\|}, \quad i = \overline{1, N-1}. \tag{S3}$$

To avoid the high-frequency noise in $\boldsymbol{e}_{\text{T}}^i$, the vector components are smoothed by a Gaussian filter. Then, the binormal vector for the set of points reads [11]

$$\boldsymbol{e}_{\text{B}}^{2i} = \boldsymbol{e}_{\text{B}}^{2i-1} = \frac{\boldsymbol{e}_{\text{T}}^{2i} \times \boldsymbol{e}_{\text{T}}^{2i+1}}{\|\boldsymbol{e}_{\text{T}}^{2i} \times \boldsymbol{e}_{\text{T}}^{2i+1}\|}, \quad i = \overline{1, N//2}, \tag{S4}$$

with // being the integer division and is also smoothed by a Gaussian filter. The normal directions are defined from (S3) and (S4) as follows

$$\boldsymbol{e}_{\mathrm{N}}^{i} = \boldsymbol{e}_{\mathrm{B}}^{i} \times \boldsymbol{e}_{\mathrm{T}}^{i}, \quad i = \overline{2, N-2}. \tag{S5}$$

It should be noted that while the tangential direction can be calculated from the boundary conditions on $\boldsymbol{m}$ [12], the discrete binormal vector, $\boldsymbol{e}_{\mathrm{B}}^{i}$, requires four points to be determined. Therefore, nodes at the ends of the vortex line are skipped in the discrete analysis.

Furthermore, the absolute values of the discrete curvature, κ_v, and torsion, τ_v, at the i-th node of the vortex string are determined via angles between the neighboring tangential and binormal unit vectors [11], respectively:

$$\kappa_v^i = \frac{(\widehat{\boldsymbol{e}_{\mathrm{T}}^{i}, \boldsymbol{e}_{\mathrm{T}}^{i+1}})}{\|\boldsymbol{\gamma}_i\|}, \tag{S6}$$

$$|\tau_v^i| = \frac{(\widehat{\boldsymbol{e}_{\mathrm{B}}^{i}, \boldsymbol{e}_{\mathrm{B}}^{i+1}})}{\|\boldsymbol{\gamma}_i\|}, \quad i = \overline{2, N-1}, \tag{S7}$$

where $\|\boldsymbol{\gamma}_i\|$ is the length of the i-th line segment. The sign of τ_v^i is determined by the rotation of the moving trihedron $\{\boldsymbol{e}_{\mathrm{T}}^{i}, \boldsymbol{e}_{\mathrm{N}}^{i}, \boldsymbol{e}_{\mathrm{B}}^{i}\}$ along $\boldsymbol{\gamma}$. Each specific pair of curvature and torsion can be associated with the radius, R_v, and pitch, P_v, of the corresponding helix formed by the vortex string as follows

$$R_v = \frac{\kappa_v}{\kappa_v^2 + \tau_v^2}, \tag{S8}$$

$$P_v = \frac{2\,\pi\,|\tau_v|}{\kappa_v^2 + \tau_v^2} \tag{S9}$$

The positive value of τ_v corresponds to the right-handed rotation, while the negative one represents the left-handed helix rotation. It should be noted that the torsion is highly sensitive to small deviations from the strictly straight string. Namely, a helix with small radius and large pitch being geometrically close to a straight line, is characterized by small κ_v and large τ_v. In the following, we will base our geometrical characterization of the vortex string for each sample geometry by average values of curvature, $\langle\kappa_v\rangle$, and torsion, $\langle\tau_v\rangle$, as well as $\langle R_v\rangle$ and $\langle P_v\rangle$.

Supplementary Note 2. Magnetic helicity of the vortex texture

We rely on the following definition of the magnetic helicity [13, 14], which normalized to its absolute value I_0 reads

$$\widetilde{C} = \frac{1}{I_0} \int\limits_V \mathrm{d}\boldsymbol{r}\, \boldsymbol{m} \cdot [\nabla \times \boldsymbol{m}] = \pm 1. \tag{S10}$$

We note, that the concept of helicity is widely used to introduce the parity break in various fields of physics including magnetohydrodynamics, superfluids, nematic systems and plasma physics [13–15]. For vortex texture, Eq. (S10) links the direction of the circulation C of the magnetization field and polarity P. We note that the circulation is defined as follows [16]

$$C = -\frac{1}{L} \int\limits_S \mathrm{d}S \boldsymbol{e}_\mathrm{n} \cdot [\nabla \times \boldsymbol{m}], \tag{S11}$$

where L is the length of a contour that bounds the region with the area S and boundary ∂S far from the vortex core, and $\boldsymbol{e}_\mathrm{n}$ is the normal to the area S, which is perpendicular to vortex string. When introduced in this way, the circulation C is nomalized to ± 1. The parameter $\widetilde{C}$ is defined to be positive if the axial vector associated with the circulation direction is collinear with the direction of magnetization along the vortex string. This magnetization is normalized to 1 and its direction is given by the sign of polarity $P = \pm 1$. It is possible to show that the expression (S10) is proportional to the product $C\,P$ for the case of the vortex in a planar disk with thickness h and radius $R \gg \ell$, the area $S = \pi R^2$. The expression $\widetilde{\widetilde{C}} = C\,P$ is typically used to discriminate four vortex states with respect to polarity and circulation [17, 18] and often refered to as handedness. Indeed, we can use the Ansatz for the magnetization $\boldsymbol{m} = \{m_x, m_y, m_z\} = \{\sqrt{1-m_z^2}\cos\phi, \sqrt{1-m_z^2}\sin\phi, m_z\}$ where $m_z = P\exp[-\rho^2/(2\ell^2)]$, $\phi = \chi + C\pi/2$ and cylindrical reference frame $\{\rho, \chi, z\}$, which allows to calculate the magnetic helicity (S10) to be $\widetilde{C} = C\,P \equiv \widetilde{\widetilde{C}}$ with the normalization coefficient $I_0 = 2\pi\,h \int_0^R \mathrm{d}\rho\, g(\rho) \left[\sqrt{1-g(\rho)^2} + \rho^2 g(\rho) \left(g(\rho) - \sqrt{1-g(\rho)^2}\right) / \left(\ell^2 \sqrt{1-g(\rho)^2}\right)\right] \approx 8.41\ell\,h$, function $g(\rho) = |m_z|$. In contrast to the vortex texture in a planar disk, the Ansatz for a vortex in an object of complex geometry can be hardly generalized. Still, for the vortex texture with an exponentially localized core, the integral in the nominator of the expression (S10) is finite and the expression can be normalized to ± 1. For instance, for the experimental geometry (Fig. 3, main text) we obtain $I_0 \approx 9955$ nm^2 for $C = +1$ and $P = +1$ (corresponding magentochirality is +1) and $I_0 \approx 12706$ nm^2 for $C = +1$ and $P = -1$ ($\widetilde{C} = -1$). We note that the normalization coefficient for vortices with opposite magnetic helicities is different due to the distinct lengths of the vortex strings (Supplementary Figure 11 and 12).

Supplementary Note 3. Influence of the sample shape on the vortex texture

A. Flat achiral disks

A spatial configuration of a magnetization texture in a confined sample is determined by the interplay between intrinsic magnetic interactions and shape (geometry) of the sample. For instance, closed-flux textures are formed in soft ferromagnetic confined samples due to the necessity for the magnetization to be parallel to the sample edge at every point along the outer normal [19, 20]. In the case of flat disks, the magnetic vortex configuration assures that the magnetization follows the sample edge through a circulating in-plane component with either counter-clockwise ($C = +1$) or clockwise ($C = -1$) magnetization rotation [1–3]. Moreover, due to the interplay between the long-range magnetostatic interaction and the short-range exchange interaction, the vortex core possesses out-of-plane magnetization component directed up ($P = +1$) or down ($P = -1$). This state is doubly degenerate with respect to the change of P and C as well as the vortex magnetic helicity $\widetilde{C} = C\,P$.

The resulting boundary effects impose restrictions on the spatial configuration of the magnetic texture. A cylinder-shaped nanodisk with flat top and bottom surfaces is an object of high symmetry possessing an inversion center i, an infinite-fold rotation axis (cylinder axis) C_∞ with a horizontal mirror plane σ_{hor}, infinite number of vertical mirror planes σ_{ver} that contains the rotation axis, and an infinite number of two-fold axes of symmetry C_2, which are perpendicular to the cylinder axis [21], see Supplementary Fig. 2a. The magnetic system in addition to the inherited geometrical mirror symmetries also have the time inversion symmetry, which has implications on geometric symmetries of the vortex state. Namely, the vertical mirror does not change the geometrical shape, but reverses the vortex polarity, while the horizontal mirror changes the vortex circulation. In both cases, vortex magnetic helicity is changed to the opposite one, see Fig. 2. Magnetic states in a nanodisk of radius $R = 150$ nm and thickness $h = 20$ nm are calculated using finite-element micromagnetic code TetraMag for material parameters of permalloy (saturation magnetization $\mu_0 M_s = 1.08$ T, exchange constant $A = 1.3 \times 10^{-11}$ J/m). Equilibrium vortex states are obtained by the conjugate gradient method. As expected, the centered magnetic vortex distribution is doubly degenerate and exhibits identical energies and vortex core dimensions independent of the vortex magnetic helicity $\widetilde{C}$, see Supplementary Fig. 2b.

B. Flat disks with DMI

For samples with the Dzyaloshinskii-Moriya interaction (DMI) [22, 23], only a certain spatial rotation direction in the magnetization distribution will be favored. In the case of the magnetic vortex distribution in a fully symmetric magnetic nanodisk, the presence of DMI leads to the elimination of vortex state degeneracy with respect to its magnetic helicity, $\widetilde{C}$, and introduces broadening (narrowing) of the vortex core region for vortices with favorable (unfavorable) magnetic helicity [24]. For the case of bulk DMI with the energy $E_{\text{DMI}} = D \int_V \mathrm{d}\boldsymbol{r}\,\boldsymbol{m} \cdot [\nabla \times \boldsymbol{m}]$ and positive DMI constant $D > 0$, it is predicted that the favorable vortex possesses the $\widetilde{C} = -1$. This is illustrated in Supplementary Fig. 2c for the case of a flat permalloy disk (radius $R = 150$ nm and thickness $h = 20$ nm) with the bulk type DMI of a constant $D = 0.2$ mJ/m^2. In this way, the bulk type DMI lifts the energy degeneracy between different vortex states, but keeps symmetry operations discussed in the Supplementary Note 3 A. The corresponding calculation is performed using MuMax3 code [25, 26]. This type of DMI favors helicoidal magnetization rotations around the tangential direction between neighboring magnetic ions. The resulting equilibrium vortex states obtained by the conjugate gradient method reveal that vortices with $\widetilde{C} = +1$ (combinations of $P = +1$, $C = +1$ and $P = -1$, $C = -1$) become metastable, as they have higher total energy than vortex states with $\widetilde{C} = -1$ (combinations of $P = +1$, $C = -1$ and $P = -1$, $C = +1$). Moreover, the size of the vortex core for the state with the magnetic helicity $\widetilde{C} = +1$ is smaller than the one for $\widetilde{C} = -1$, see Supplementary Fig. 2c. Further discussion on the impact of the intrinsic DMI on the vortex texture can be found in Ref. [24].

C. Achiral asymmetric nanodot with axial symmetry

For an intrinsically isotropic achiral ($D = 0$) ferromagnetic material, the elimination of a certain geometric symmetry leads to a change of the equilibrium magnetic texture. Axially symmetric modification of the top surface of the nanodisk like a Gaussian bump lifts the horizontal mirror plane and two-fold rotation axes. The symmetry of magnetic vortex depends on the lateral size of the sample. For a moderate disk thickness and bump height, we observe that the vortex string, being straight in the flat sample, possesses a bend with curling, see Fig. 3a. Full-scale micromagnetic

simulations performed for an asymmetric nanodisk ($R = 150$ nm and $h = 15$ nm) with a centered Gaussian bump ($t = 20$ nm, $x_0 = 0$ nm and $b = 20$ nm) reveal the presence of vortices with two different homochiral deformations of the vortex string, see Supplementary Fig. 3b. Namely, vortex strings of vortices with different magnetic helicity, $\widetilde{C}$, acquire a homochiral deformation: the resulting curling direction of the vortex string is right-handed for vortices with $\widetilde{C} = +1$ and left-handed for vortices with $\widetilde{C} = -1$. Moreover, the equilibrium vortex strings can take any angular position around the center axis of the nanodisk, whose locus forms a smooth surface of stable vortex positions, which corresponds to an infinite manifold of vortex states, see Supplementary Fig. 3c and d. Tall but narrow enough bump pins the vortex. If the thickness of the disk below the bump is large enough, vortex string can obtain a small bend, see also discussion in Supplementary Note 4. Thus, the magnetic state in the discussed geometry can preserve the only vertical mirror plane m' to keep the magnetic helicity $\widetilde{C}$.

D. Achiral asymmetric nanodisk with reflection symmetry

Here, we consider a sample with an off-centered Gaussian bump in contrast to the system with axial symmetry considered in Supplementary Note 3 C. In this case, sample's geometry keeps only the vertical mirror plane, see Supplementary Fig. 3e. This reduces the number of equilibrium vortex string positions from an infinite manifold (as was discussed for the case of an asymmetric nanodisk with a centered Gaussian bump) to only one stable angular position near the bump for each of the vortex magnetic helicity $\widetilde{C}$. Namely, the equilibrium vortex strings for $\widetilde{C} = +1$ and $\widetilde{C} = -1$ remain in the center region of the nanodisk and obtain homochiral curling deformation being right- ($\tau_v > 0$) and left-handed ($\tau_v < 0$), respectively. This is confirmed by means of full-scale micromagnetic simulations for an asymmetric nanodisk ($R = 150$ nm and $h = 15$ nm) with an off-centered Gaussian bump on its top surface ($t = 20$ nm, $x_0 = 10$ nm and $b = 20$ nm), see Supplementary Fig. 3f. The resulting vortex strings for $\widetilde{C} = +1$ and $\widetilde{C} = -1$ obtain the same total energy but occupy different angular positions with respect to the symmetry plane $\hat{\boldsymbol{x}}\hat{\boldsymbol{z}}$, see Supplementary Fig. 3g. Namely, the vortex string of the vortex with $\widetilde{C} = +1$ intersects the plane $\hat{\boldsymbol{x}}\hat{\boldsymbol{z}}$ at $\psi < 0$, while the string with $\widetilde{C} = -1$ crosses it at $\psi > 0$. It should be also noted that in contrast to the case of a nanodisk with a centered Gaussian bump, where vortex strings could obtain any angular position, in the case of a disk with an off-centered bump, vortices on the far side of the Gaussian bump become unstable and are not observed in micromagnetic simulations, see Supplementary Fig. 3h. Thus, the elimination of high symmetries of the sample geometry leads not only to the elimination of the topological degeneracy but also introduces magnetic symmetry breaking effect, which results in the appearance of the homochiral deformation of the vortex string. The symmetry of the magnetic state becomes of the lowest one in the absence of mirror planes and axes of rotation.

Supplementary Note 4. Phase diagram of magnetic states

To analyse the influence of the surface, ς, and volume, λ, magnetostatic charges on the formation of curved vortex strings in asymmetric nanodisks, we perform full-scale finite element micromagnetic simulations and compute phase diagrams of the integral surface and volume magnetostatic charges Σ (Supplementary Fig. 5a) and Λ (Supplementary Fig. 5b), respectively. Change of the magnetic geometry from the symmetric to the asymmetric nanodisk is parameterized by means of the surface asymmetry ratio $\zeta^{\text{A}} = (S^{\text{T}} - S^{\text{B}}) / (S^{\text{T}} + S^{\text{B}})$, which characterizes the difference between the top and bottom surface areas S^{T} and S^{B}, respectively, and effective thickness, $\langle h \rangle = 1/S^{\text{B}} \int z\mathrm{d}\boldsymbol{r}$. In the frame of this parametrization, symmetric magnetic nanodisks have $\zeta^{\text{A}} = 0$ % and $\langle h \rangle = h$, while asymmetric ones have different range of geometric parameters presented in Supplementary Fig. 4. In the case of the vortex state in a symmetric flat nanodisks, the total volume magnetostatic charge is absent ($\Lambda = 0$) due to the formation of the flux-closed magnetization distribution (Supplementary Fig. 5b). The surface magnetostatic charges are present only on the top and bottom surfaces in the center region of the nanodisk, where the out-of-plane magnetization component of the vortex core is localized. Moreover, being the same by the absolute value but having different sign, they compensate each other resulting in $\Sigma = 0$ (Supplementary Fig. 5a).

Breaking the sample's symmetry by an off-centered Gaussian bump leads to the homochiral spatial deformation of the vortex string. We observe this effect in simulations and discuss it in the following for a Gaussian bump offset by $q = 10$ nm. Curled vortex strings produce uncompensated total surface and volume magnetostatic charges, see Supplementary Fig. 5a and b, respectively. Thus, as they are non-zero, there is a broad range of geometric parameters $\langle h \rangle$ and ζ^{A}, where their product is negative (Supplementary Fig. 5c). This forces the micromagnetic system to optimize the vortex string structure at equilibrium by introducing homochiral bending of the vortex string.

For highly asymmetric $\zeta^{\text{A}} \gtrsim 2$ % yet ultrathin nanodots (sample with $x_0 = 10$ nm, $t = 50$ nm, $b = 20$ nm and $h = 5$ nm), the sample acquires stabilization of the nearly straight vortex string with $\langle \kappa_v \rangle = 0.7\ \mu\text{m}^{-1}$ and $\langle \tau_v \rangle = 3.7\ \mu\text{m}^{-1}$) pinned near the apex region of the Gaussian bump, see Supplementary Fig. 6a. Such pinning originates from the shape anisotropy along $\hat{\boldsymbol{z}}$ direction introduced by uncompensated surface magnetostatic charges on the top and bottom surfaces [27]. Thus, the resulting vortex configuration introduces a significant integral volume, Σ, and surface, Λ, magnetostatic charges, see Supplementary Fig. 5a and b. The resulting vortex string is mainly straight with the appearance of small bending in the middle part, see Supplementary Fig. 6b and c, which is reflected in the nearly zero vortex curvature (Supplementary Fig. 6d) but prominent torsion (Supplementary Fig. 6e). The respective vortex string can be represented as the helix with the radius, $\langle R_v \rangle = 9.1\ell$, and pitch, $\langle P_v \rangle = 308.2\ell$. It should be noted that the vortex string in such highly asymmetric nanodots do not cross the mirror symmetry plane $\hat{\boldsymbol{x}}\hat{\boldsymbol{z}}$.

In a thin asymmetric nanodisk with comparatively small effective thickness $\langle h \rangle \lesssim 3\ell$ (sample with $h = 15$ nm accommodating a Gaussian bump ($x_0 = 10$ nm, $t = 20$ nm, $b = 10$ nm)), the vortex core is shifted outside the bump area towards the disk center being opposite to the bump region, see Supplementary Fig. 7a. This state corresponds to the primary optimization of the exchange energy by the minimization of the vortex string length at the thinnest flat part of the sample, while the energy loss in exchange due to the asymmetry of the vortex texture is small according to the measure of shift from the disk center. The magnetostatics mainly acts on the texture via the surface magnetostatic charges, assuring the in-plane shape anisotropy in the vicinity of the vortex string. For such samples, we observe weakly curved (Supplementary Fig. 7b and c) vortex strings with $\langle \kappa \rangle = 6.7\ \mu\text{m}^{-1}$ and $\langle \tau \rangle = 3.0\ \mu\text{m}^{-1}$, see Supplementary Fig. 7d and e. For the latter, average string radius, $\langle R_v \rangle$, and pitch, $\langle P_v \rangle$, are about 24.1ℓ and 60.4ℓ, respectively.

Thick enough samples with $\langle h \rangle \gtrsim 3\ell$ and noticeable asymmetry in top and bottom surfaces ($\zeta^{\text{A}} \lesssim 2$ %) support strongly bent vortex strings, see Supplementary Fig. 8a, b and c. In the particular case of the $h = 30$ nm thick sample accommodating Gaussian bump ($x_0 = 10$ nm, $t = 40$ nm, $b = 20$ nm), the curvature and torsion of the vortex string reach $\langle \kappa_v \rangle = 4\ \mu\text{m}^{-1}$ and $\langle \tau_v \rangle = 3.3\ \mu\text{m}^{-1}$, see Supplementary Fig. 8d and e. In samples of this series, there is a pronounced tendency to increase the local value of curvature along the vortex string moving from the bottom to the top surface, which is complemented by a slight increase in torsion, see Supplementary Fig. 8d,e. The respective vortex string can be represented as the helix with the radius $\langle R_v \rangle = 27.6\ell$, and pitch $\langle P_v \rangle = 144.7\ell$. The tilt of the vortex string with respect to the symmetry plane $\hat{\boldsymbol{x}}\hat{\boldsymbol{z}}$ is $\psi = -64°$ with the sign being opposite to the sign of the vortex magnetic helicity, $\widetilde{C} = +1$.

Supplementary Note 5. Influence of exchange and magnetostatic interactions on the formation of curved vortex strings

To understand the role of different magnetic interaction and their influence on the shape of the vortex string, we perform additional micromagnetic simulations using the MAGPAR program [28, 29] and of disks with an asymmetric bump of $x_0 = 10$ nm, $t = 20$ nm, $b = 20$ nm and $h = 30$ nm as a case study. The reference magnetization distribution is obtained from the full-scale micromagnetic simulations with exchange and magnetostatic interactions, see Supplementary Fig. 9a. The resulting vortex string obtains a substantial bending with $\langle\kappa_v\rangle = 6.2\ \mu\text{m}^{-1}$ and $\langle\tau_v\rangle = 9.0\ \mu\text{m}^{-1}$ (Supplementary Fig.9b–e), which corresponds to the appearance of wire of a helix shape with $\langle R_v\rangle = 9.7\ell$ and $\langle P_v\rangle = 89.5\ell$ crossing the symmetry plane at angle $\psi = -51°$, see Supplementary Table I.

In the first model case, there is only exchange interaction and pinned surface magnetization taken from the vortex distribution of the reference full-scale simulation. The resulting vortex string has $\langle\kappa_v\rangle = 2.0\ \mu\text{m}^{-1}$, which is three times smaller than the one obtained for the full-scale simulation, while the vortex string torsion $\langle\tau_v\rangle = -6.6\ \mu\text{m}^{-1}$ is opposite to the case of the full-scale simulation (Supplementary Fig. 9f–j). We note that the local vortex core dimensions increase in the absence of the magnetostatic interaction, while the vortex string is helix with $\langle R_v\rangle = 7.9\ell$ and $\langle P_v\rangle = 164.3\ell$, see Supplementary Table I.

As the surface magnetostatic charges lead to the appearance of in-plane shape-induced anisotropy for thin films, it is instructive to analyse their influence on the formation of bent vortex strings. We perform the same simulations with constrained magnetization at all sample boundaries with (i) homogeneous in-plane anisotropy with the hard axis of magnetization along $\hat{\boldsymbol{z}}$, see Supplementary Fig. 9k–o, and (ii) spatially varying "in-surface" anisotropy with the easy surface of magnetization linearly changing with $z \in [0, h]$ from the horizontal plane at the bottom sample's surface to the manifold coinciding with the top surface at $z = h$, see Supplementary Fig. 9p–t. In both these cases, we obtain similar curvatures being $\langle\kappa_v^{(\text{i})}\rangle = 3.2\ \mu\text{m}^{-1}$ and $\langle\kappa_v^{(\text{ii})}\rangle = 3.3\ \mu\text{m}^{-1}$, while torsion values are different being $\langle\tau^{(\text{i})}\rangle = 4.3\ \mu\text{m}^{-1}$ and $\langle\tau^{(\text{ii})}\rangle = 1.3\ \mu\text{m}^{-1}$, see Supplementary Table I. The cross-cut dimensions of the vortex core in both cases are substantially reduced in comparison with the ones obtained in full-scale simulations, see Supplementary Fig. 9. The distribution of curvature along the vortex string decreased from the bottom sample's surface to the top one (Supplementary Fig. 9n and s), which is in contrast with the full-scale simulations (Supplementary Fig. 9d). If we keep pinned only the magnetization at the side face of the sample in the vortex distribution, the vortex leaves the bump area to minimize the string length for both anisotropy models (i) and (ii).

If the vortex position at the top and bottom sample surfaces is not fixed manually, in absence of the full-scale magnetostatics, the vortex minimizes its length even losing part of the exchange energy due to the perturbation of the symmetric circulating texture. This allows to conclude, that the in-plane shape anisotropy coming from the surface magnetostatic charges is not responsible for the vortex positioning in thick samples. Furthermore, volume magnetostatic charges determine the vortex string position. Having the vortex in a region with substantially different top and bottom surfaces, the exchange boundary conditions force the vortex string to have a finite curvature. Its distribution along the string is determined by the exchange interaction. We stress that both, the sign of the vortex string torsion $\langle\tau_v\rangle$ and the tilt angle ψ with respect to the available plane of symmetry are dependent on $\widetilde{C}$ and volume magnetostatic charges.

Supplementary Table I and Supplementary Fig. 9 summarize the data for the sample parameters, which are discussed in the previous paragraphs, taking into account different vortex magnetochiralities. We note that the accuracy for $\langle\kappa_v\rangle$ is about 20%, while the torsion should be rather considered as an order of magnitude estimate. Magnetic textures with the same value of the vortex magnetic helicity $\widetilde{C}$ have close parameters of the vortex strings. The change of $\widetilde{C} = +1$ to $\widetilde{C} = -1$ reduces the average curvature and changes the sign of torsion. In all cases, absence of the full-scale magnetostatics substantially reduces $\langle\kappa_v\rangle$. In Supplementary Fig. 9, the distributions of the flux of the normalized topological charge $\widetilde{\Omega}$ are compared across the models discussed above. In the absence of magnetostatics, the vortex line becomes almost straight between the pinning sites. In both anisotropy models that mimic surface magnetostatic charges, an instability of the pinned configuration is also pronounced in the thickness of the Bloch line. The overall tilt angle ψ, of the vortex string with respect to the mirror symmetry plane $\hat{\boldsymbol{x}}\hat{\boldsymbol{z}}$ is found around the crossing point of the string. For all simulation models, these angles are provided in the last column of Supplementary Table I. As it follows from the sign of ψ, the change of the vortex magnetic helicity $\widetilde{C}$ changes the sign of the tilt angle of the vortex string to the opposite one.

Supplementary Note 6. Vortex states in experimental geometry

A. Supplementary Movie 1

The movie shows a 3D volume rendering of a permalloy cap (shown in the front at the starting frame) located on a permalloy-covered carbon support (shown in the back at the starting frame). The color-coded volume rendering visualizes the reconstructed density, which is proportional to the attenuation coefficient caused by the scattering absorption of the objective lens aperture in the transmission electron microscope (TEM). The voxel size is $0.5 \times 0.5 \times 0.5$ nm^3. The animation starts with a 360° rotation around the vertical axis, followed by a second 360° rotation with additional visualization of the segmented surface. The movie was created using the Avizo software package (ThermoFisher Scientific Company).

B. Spatial 3D investigation of the asymmetric permalloy cap

Supplementary Fig. 10 depicts structural features of an asymmetric permalloy cap after deposition of a 50-nm-thick permalloy film on top of a polystyrene sphere with a diameter of 80 nm.

C. Vortex strings of different magnetic helicity

To analyse the vortex texture in experimentally-determined 3D volume of the permalloy nanocap, we perform full-scale micromagnetic simulations and apply the same vortex string determination procedure as described in the Supplementary Note 1, using five different meshes for each pair of C and P, see Supplementary Fig. 11a. The resulting strings for all possible combination of vortex parameters show noticeable bending deformations, that are summarized in Supplementary Table II and shown in Supplementary Figs. 11, 12, 13 and 14. Namely, for both cases of $\widetilde{C} = +1$ ($C = +1$, $P = +1$ and $C = -1$, $P = -1$) the curvature of the vortex string increases from bottom surface of the nanocap to the top one with $\langle \kappa_v \rangle = 2.5$ μm^{-1}, see Supplementary Fig. 11e and Supplementary Fig. 14e, while τ_v remains constant along the string with $\langle \tau_v \rangle = 11 \div 12$ μm^{-1}, see Supplementary Fig. 11f and 14f. Such parameters correspond to the helical wire with radius $\langle R_v \rangle = 3.6 \div 3.9\ell$ and pitch $\langle P_v \rangle = 95.5 \div 106.0\ell$.

In the case of $\widetilde{C} = -1$ ($C = +1$, $P = -1$ and $C = -1$, $P = +1$), the curvature distribution along the vortex string increases from both surfaces of the nanocap to the middle part of it with the average value being 4.0 μm^{-1}, see Supplementary Fig. 12e and 13e. The reconstructed value of torsion for $\widetilde{C} = -1$ is small and $\langle \tau_v \rangle = 2 \div 3$ μm^{-1}, that corresponds to a slightly curved helical string with $\langle R_v \rangle = 27.5 \div 38.4\ell$ and $\langle P_v \rangle = 76.65 \div 125.4\ell$. We note that these vortex strings are longer and have different spatial localization than those obtained for $\widetilde{C} = +1$.

D. Influence of the cap support on the vortex texture

To determine the influence of the supporting part of the experimental geometry on the stabilization of vortex strings with curling deformation, we performed additional full-scale micromagnetic simulations for the truncated permalloy cap using five different meshes. The resulting vortex states for all possible sets of P and C are obtained by means of the conjugate gradient method and analysed using the procedure described in Supplementary Note 1. The geometrical parameters of the strings are summarized in Supplementary Table III and presented in Supplementary Fig. 15, 16, 17 and 18. For both cases of the vortex magnetic helicity being $\widetilde{C} = +1$ ($C = +1$, $P = +1$ and $C = -1$, $P = -1$) and $\widetilde{C} = -1$ ($C = +1$, $P = -1$ and $C = -1$, $P = +1$), the curvature of the vortex string is reduced from bottom surface to the top one with the averaged value being $\langle \kappa_v \rangle = 2.0$ μm^{-1}. Meanwhile, the torsion of the vortex string is present only for $\widetilde{C} = +1$ being $\langle \tau_v \rangle = 8$ μm^{-1}. For the case of $\widetilde{C} = -1$, torsion is near zero with $\langle \tau_v \rangle = -0.1$ μm^{-1} for $C = -1$, $P = +1$ and $\langle \tau_v \rangle = 0.2$ μm^{-1} for $C = +1$, $P = -1$. The corresponding helical string approximation for the numerically determined vortex strings are $\langle R_v \rangle = 6.0\ell$, $\langle P_v \rangle = 134.4 \div 134.8\ell$ for $\widetilde{C} = +1$ and $\langle R_v \rangle = 77.2 \div 77.4\ell$, $\langle P_v \rangle = 9.4 \div 23.5\ell$ for $\widetilde{C} = -1$.

The resulting geometrical parameters of the vortex strings for the truncated cap are found to be quantitatively different from ones obtained for the full cap geometry. Still the resulting qualitative dependencies of $\langle R_v \rangle$ and $\langle P_v \rangle$ for vortex strings in both cases of full and truncated caps indicate the presence of chiral asymmetry in the observed vortex strings. Thus, the main influence on the appearance of non-local chiral effects arises from the nanocap itself and its asymmetrical shape. We note that the resulting positions of the vortex strings for the truncated cap are

different from ones obtained for the full cap geometry, which indicates on the reduced influence of the magnetostatic interaction on the flux-closed vortex magnetic texture.

Supplementary Note 7. Principal directions and principal curvatures of a curvilinear shell

Any two-dimensional curvilinear surface embedded in the Cartesian frame of reference $\{\hat{\boldsymbol{x}}, \hat{\boldsymbol{y}}, \hat{\boldsymbol{z}}\}$ can be parametrized as

$$\boldsymbol{r}(\xi_1, \xi_2) = r_x(\xi_1, \xi_2)\,\hat{\boldsymbol{x}} + r_y(\xi_1, \xi_2)\,\hat{\boldsymbol{y}} + r_z(\xi_1, \xi_2)\,\hat{\boldsymbol{z}}, \tag{S12}$$

where ξ_1 and ξ_2 are curvilinear coordinates on the surface. At each point of the surface, it is possible to introduce tangential vectors

$$\boldsymbol{g}_\alpha = \partial_\alpha \boldsymbol{r}(\xi_1, \xi_2), \qquad \partial_\alpha = \frac{\partial}{\partial \xi_\alpha}, \tag{S13}$$

with $\alpha = 1, 2$. The vectors $\boldsymbol{g}_1$ and $\boldsymbol{g}_2$ determine the tangent plane to the surface and the normal vector

$$\boldsymbol{n} = \frac{\boldsymbol{g}_1 \times \boldsymbol{g}_2}{|\boldsymbol{g}_1 \times \boldsymbol{g}_2|}. \tag{S14}$$

The way of measuring distances on a curved surface is defined by the metric tensor or the first fundamental form, which is a second rank symmetric tensor

$$\left\| g_{\alpha\beta} \right\| = \begin{Vmatrix} g_{11} & g_{12} \\ g_{21} & g_{22} \end{Vmatrix}, \qquad g_{\alpha\beta} = \boldsymbol{g}_\alpha \cdot \boldsymbol{g}_\beta, \tag{S15}$$

This metric tensor determines an element of the surface area and reads

$$\mathrm{d}S = \sqrt{g}\,\mathrm{d}\xi_1\,\mathrm{d}\xi_2, \qquad g = \det \left\| g_{\alpha\beta} \right\| = |\boldsymbol{g}_1 \times \boldsymbol{g}_2|^2. \tag{S16}$$

The second fundamental form or shape tensor reads

$$\left\| b_{\alpha\beta} \right\| = \begin{Vmatrix} b_{11} & b_{12} \\ b_{21} & b_{22} \end{Vmatrix}, \qquad b_{\alpha\beta} = \boldsymbol{n} \cdot \partial_\beta \boldsymbol{g}_\alpha. \tag{S17}$$

It should be noted that the first fundamental form determines the so-called intrinsic geometry of the surface (S12) and permits the calculation of its curvature and metric properties. In turn, the second fundamental form characterizes the so-called extrinsic geometry of the surface and shows the way how the curved surface is embedded in a surrounding space. Together the first and second fundamental forms allow to determine local geometric invariants of the surface by the definition of the shape operator

$$\left\| S_{\alpha\beta} \right\| = \frac{1}{g} \begin{Vmatrix} b_{11}\, g_{22} - b_{12}\, g_{12} & b_{12}\, g_{22} - b_{22}\, g_{12} \\ b_{12}\, g_{11} - b_{11}\, g_{12} & b_{22}\, g_{11} - b_{12}\, g_{12} \end{Vmatrix}. \tag{S18}$$

While the eigenvalues of (S18) determine the principal curvatures $\kappa_{1,2}$ of the surface (S12), the normalized eigenvectors of the shape operator define the orthogonal basis $\boldsymbol{e}_{1,2}$. Vectors $\boldsymbol{e}_{1,2}$ point out along the principal directions, along which the surface curvatures obtain their extreme values $\kappa_{1,2}$, see Supplementary Fig. 19a. It should be noted that principal curvatures are geometric invariants of the surface, because they correspond to the maximal and minimal values of the curvature in any point on the surface, see Supplementary Fig. 19b,c. While the product of the principal curvatures determines the Gaussian curvature $K = \kappa_1\,\kappa_2$, see Supplementary Fig. 19d, their mean value $H = (\kappa_1 + \kappa_2)/2$ is called the mean curvature, see Supplementary Fig. 19e.

In the case of discrete curved surfaces based on triangular meshes, the estimation of the principal curvatures and principal directions is based on the finite-difference scheme proposed in Ref. [30]. This method relies on the tensor of curvature that assigns each point $P(\boldsymbol{r})$ of a curved surface to the function that measures the curvature $\kappa(\boldsymbol{t})$ at $P(\boldsymbol{r})$ along the unit vector $\boldsymbol{t}$, which is tangential to the surface.

Supplementary Figures

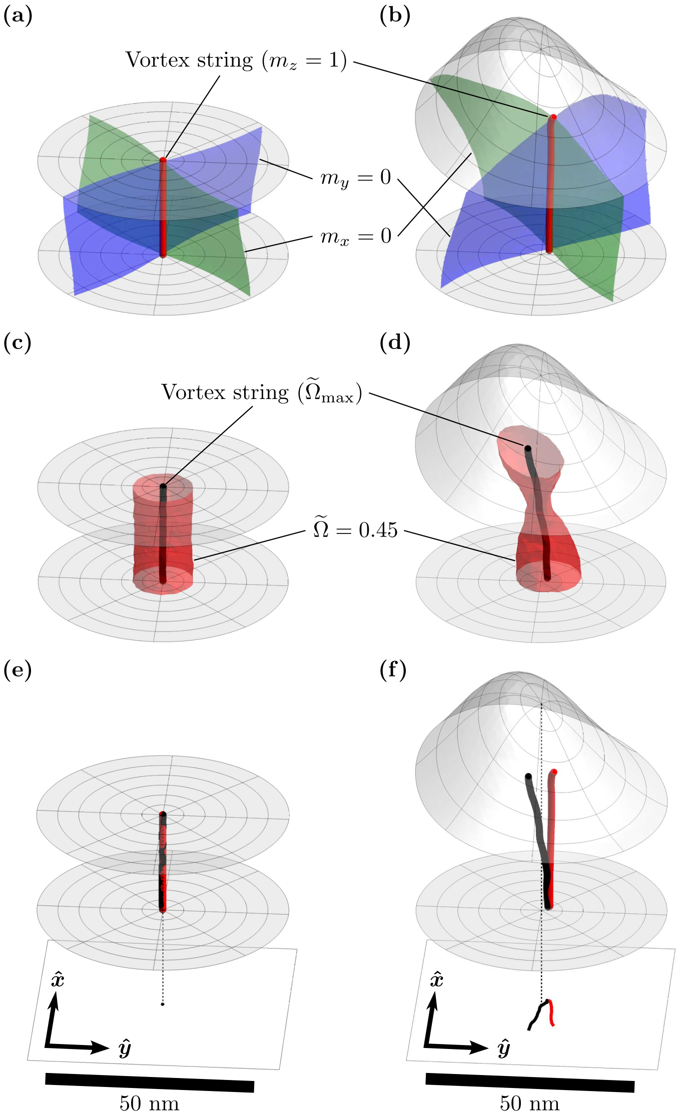

Supplementary Fig. 1. **Comparison of methods for the vortex string determination for symmetric and asymmetric nanodisks.** **(a)** and **(b)** show the vortex string (red lines, $m_z = 1$) determined by means of the intersection between isosurfaces $m_x = 0$ (green) and $m_y = 0$ (blue) for a flat symmetric nanodisk and curved asymmetric nanodisk, respectively. Both nanodisks are of radius $R = 150$ nm and thickness $h = 30$ nm. The asymmetric nanodisk has an off-centered Gaussian bump with a height $t = 40$ nm, width $b = 20$ nm and lateral shift $x_0 = 10$ nm on the top surface. **(c)** and **(d)** depict the reconstruction of the vortex strings (black lines, $\widetilde{\Omega}_{\max}$) from the maximum of the normalized flux density distribution of the topological charge $\widetilde{\Omega}$ for symmetric and asymmetric nanodisks, respectively. The red region depicts the distribution of the $\widetilde{\Omega} = 0.45$ for both geometries. **(e)** and **(f)** show the comparison of the location of vortex strings and their projections on the XY plane determined based on the two methods, respectively. We note that in **(a-f)** we show only a selected part of the nanodisk around the vortex string.

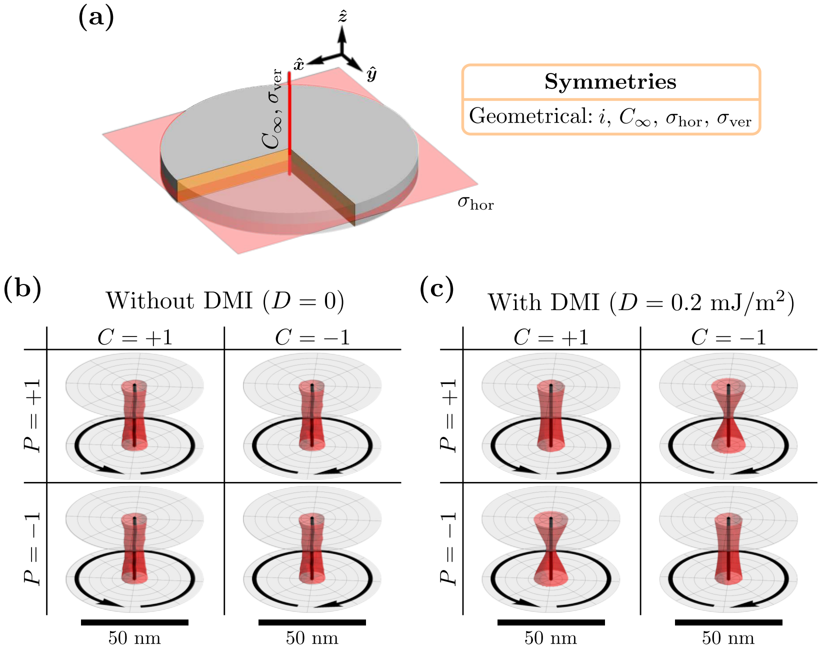

Supplementary Fig. 2. **Vortex states in symmetric flat nanodisks with and without DMI of bulk type.** **(a)** Schematic image of a flat symmetric nanodisk and point symmetry groups associated with the geometry of the sample. Diagrams of equilibrium vortex states in a nanodisk of radius $R = 150$ nm and thickness $h = 20$ nm **(b)** without and **(c)** with bulk DMI possessing the DMI constant $D = 0.2$ mJ/m^2. The presence of DMI lifts the double degeneracy of the vortex state with respect to the vortex magnetic helicity. The energetically favourable vortex state has the magnetic helicity $\widetilde{C} = C\,P = -1$, for the chosen type of DMI and the sign of the DMI constant, while the unfavorable vortex is characterized by $\widetilde{C} = +1$. The twist due to the intrinsic DMI on the vortex texture leads to the modification of the vortex core size. Further analysis of this effect is given by Butenko *et al.* [24]. In the diagrams shown in panels **(b,c)** we show only a center part of the nanodisk with diameter of 60 nm around the vortex string (black curve). Red regions shown in panels **(b,c)** indicate the distribution of the normalized topologically flux density $\widetilde{\Omega} = 0.8$ around the vortex string.

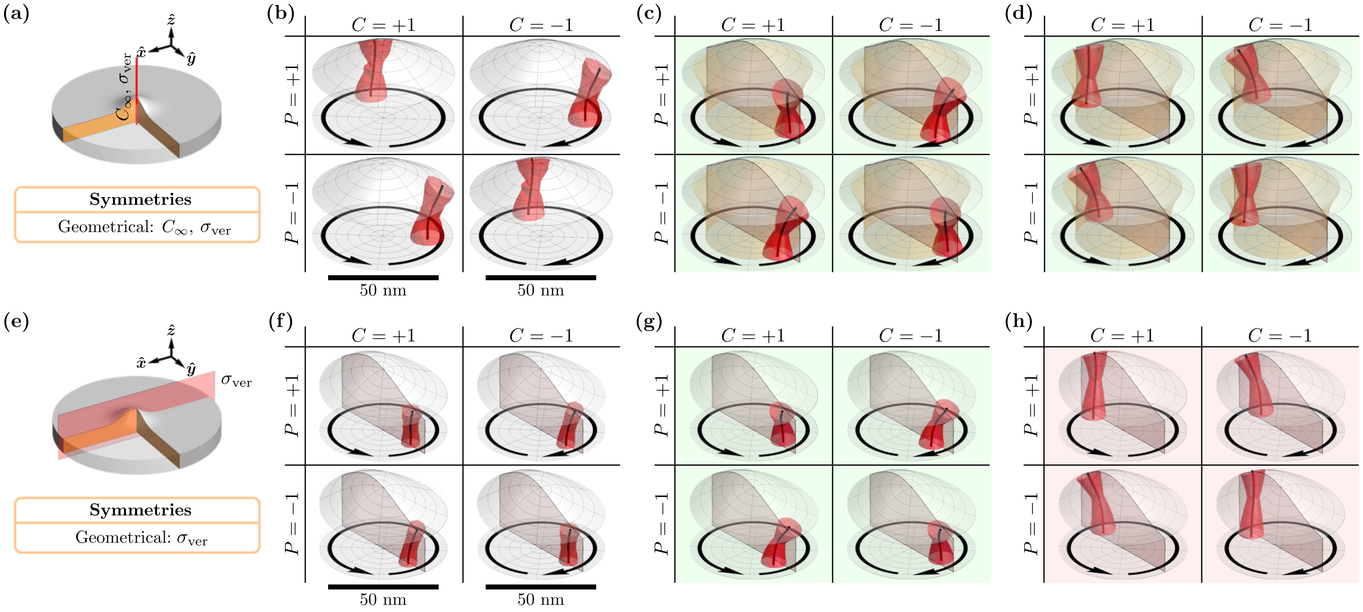

Supplementary Fig. 3. **Vortex states in asymmetric nanodisks with and without axial symmetry.** **(a)** Schematic image of an asymmetric nanodisk with centered Gaussian bump and indicated point symmetry groups associated with the geometry of the sample. **(b)** Diagrams of equilibrium vortex states obtained by means of full-scale micromagnetic simulations for an asymmetric nanodisk of radius $R = 150$ nm, thickness $h = 15$ nm and a centered Gaussian bump ($t = 20$ nm, $x_0 = 0$ nm and $b = 20$ nm). The black line indicates the vortex string position. Vortex string obtains a homochiral deformation dependent on the sign of the vortex magnetic helicity, $\widetilde{C} = CP$. **(c)** and **(d)** show schematic state diagrams depicting stable (filled with green) equilibrium vortex states of different vortex circulation C and polarity P on the Gaussian bump at different positions with respect to the symmetry plane. The equilibrium vortex strings can take any angular position around the center axis of the nanodisk. The locus of all angular positions of stable vortex states forms a smooth surface (indicated in brown), which corresponds to an infinite manifold of vortex states. **(e)** Schematic image of an asymmetric magnetic nanodisk with off-centered Gaussian bump. The only remaining geometric mirror symmetry is S_2^{xz}. **(f)** Diagrams of equilibrium vortex states obtained for an asymmetric nanodisk ($R = 150$ nm and $h = 15$ nm) with an off-centered Gaussian bump ($t = 20$ nm, $x_0 = 10$ nm and $b = 20$ nm). The resulting vortex strings possess bending deformations with a homochiral curling specific to $\widetilde{C}$. Namely, vortex strings for $\widetilde{C} = +1$ and $\widetilde{C} = -1$ are crossing the $\hat{\boldsymbol{x}}\hat{\boldsymbol{z}}$ plane from different sides, but at the same angle $|\psi|$. **(g)** Stable (filled with green) and **(h)** unstable (filled with red) vortex states depend on the angular position of the vortex string around the Gaussian bump. Only those vortices, which are bent towards the center of the disk are stable, see panel **(g)**. Those vortices, which are bent towards the edge of the disk are unstable, see panel **(h)**. In the diagrams **(b-d)** and **(f-h)** we show only a selected part of the nanodisk around the center of the Gaussian bump. Red regions in all diagrams **(b)** - **(d)** and **(f)** - **(h)** show locus of points $\widetilde{\Omega} = 0.6$.

(a)

#	q, nm	t, nm	b, nm	h, nm	$\langle h\rangle/\ell$	ζ^{A}, %
1	0	0	0	5	0.9	0
2	10	10	10	5	1	0.11
3	10	20	10	5	1	0.37
4	10	30	10	5	1	0.73
5	10	40	20	5	1	1.47
6	10	50	20	5	1	2.14
7	10	60	20	5	1	2.85
8	0	0	0	10	1	0
9	10	10	10	10	1	0.11
10	10	20	10	10	1	0.37
11	10	30	10	10	1	0.73
12	10	40	20	10	2	1.47
13	10	50	20	10	2	2.13
14	10	60	20	10	2	2.85
15	10	10	10	15	2	0.11
16	10	20	10	15	2	0.37
17	10	30	10	15	2	0.73
18	10	40	20	15	3	1.47
19	10	50	20	15	3	2.13
20	10	60	20	15	3	2.85
21	0	0	0	20	3	0
22	10	10	10	20	3	0.11
23	10	20	10	20	3	0.37
24	10	30	10	20	3	0.73
25	10	40	20	20	4	1.47
26	10	50	20	20	4	2.13
27	10	60	20	20	4	2.85
28	0	0	0	30	5	0
29	10	10	10	30	5	0.11
30	10	20	10	30	5	0.37
31	10	30	10	30	5	0.73
32	10	40	20	30	5	1.47
33	10	50	20	30	6	2.13
34	10	10	10	40	7	0.11
35	10	20	10	40	7	0.37
36	10	30	10	40	7	0.73
37	10	40	20	40	7	1.47
38	10	50	20	40	7	2.13
39	10	60	20	40	8	2.85

(b)

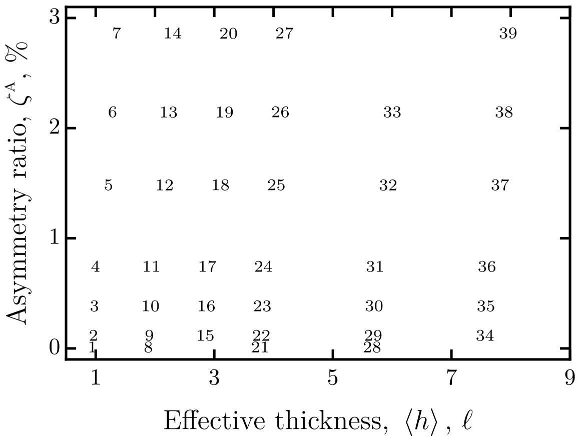

Supplementary Fig. 4. **Geometrical parameters of symmetric and asymmetric nanodisks used for the calculation of phase diagrams**. **(a)** Geometrical parameters for the geometries studied in simulations to find surface and volume magnetostatic charges. **(b)** Graphical representation of the data shown in panel (a). This representation corresponds to the Fig. **2** of the main text and Supplementary Fig. 5.

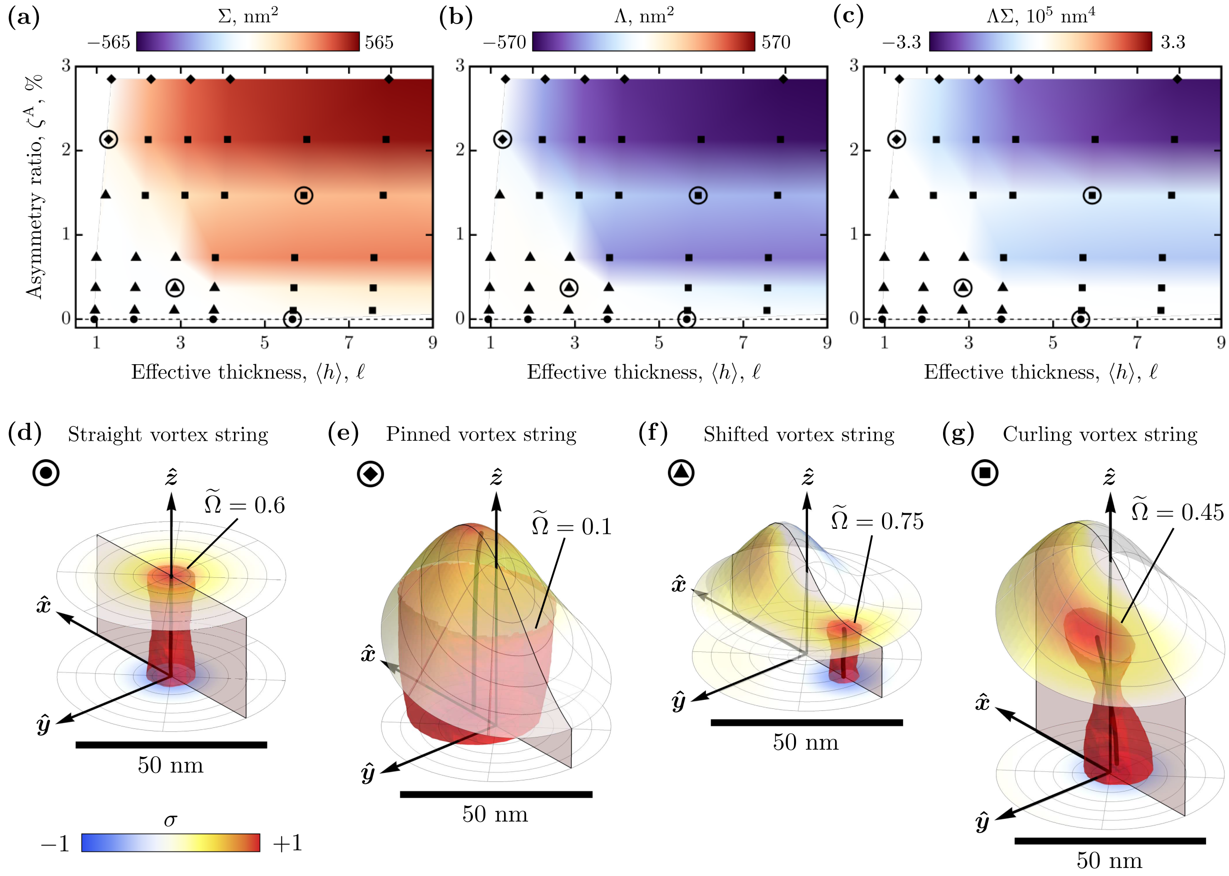

Supplementary Fig. 5. **Phase diagrams of integral magnetostatic charges for different equilibrium magnetization states in symmetric and asymmetric achiral ($D = 0$) nanodisks.** The distribution of the normalized integral surface Σ, **(a)**, and volume Λ, **(b)**, magnetostatic charges as well as their product $\Sigma\Lambda$, **(c)**, dependent on the asymmetry ratio, ζ^{A}, and the effective average thickness, $\langle h\rangle$, of a magnetic nanodisk. Symbols on the phase diagrams correspond to the results of full-scale micromagnetic simulations of symmetric and asymmetric magnetic nanodisks with a radius of 150 nm. The type of symbol indicates features of the vortex state: **(d)** Circle corresponds to the vortex state in a symmetric nanodisk with a thickness of 5.5ℓ ($h = 30$ nm). Here, the vortex string is a straight line. Colormap indicates the distribution of the surface magnetostatic charges on the top and bottom surfaces. **(e)** Diamond indicates the state in a highly asymmetric nanodisks of small thickness of about ℓ ($h = 5$ nm) and a tall off-centered Gaussian bump ($t = 50$ nm, $b = 20$ nm and $x_0 = 10$ nm). The vortex string in this object is pinned at the apex of the Gaussian bump. **(f)** Triangle indicates the state in a thicker nanodisk ($h = 15$ nm $\approx 3\ell$) with an off-centered shallower Gaussian bump ($t = 20$ nm, width $b = 10$ nm and shift $x_0 = 10$ nm). The vortex string in this object is shifted aside from the Gaussian bump to the flat part of the disk. **(g)** Square indicates the state in a thick nanodisk possessing a pronounced asymmetry due to the presence of a Gaussian bump (geometry of the object: $h = 30$ nm, $t = 40$ nm, $b = 20$ nm and $x_0 = 10$ nm). In this case, the vortex string obtains strong bending with the additional homochiral curling deformation, which direction is dependent on the vortex magnetic helicity $\widetilde{C}$.

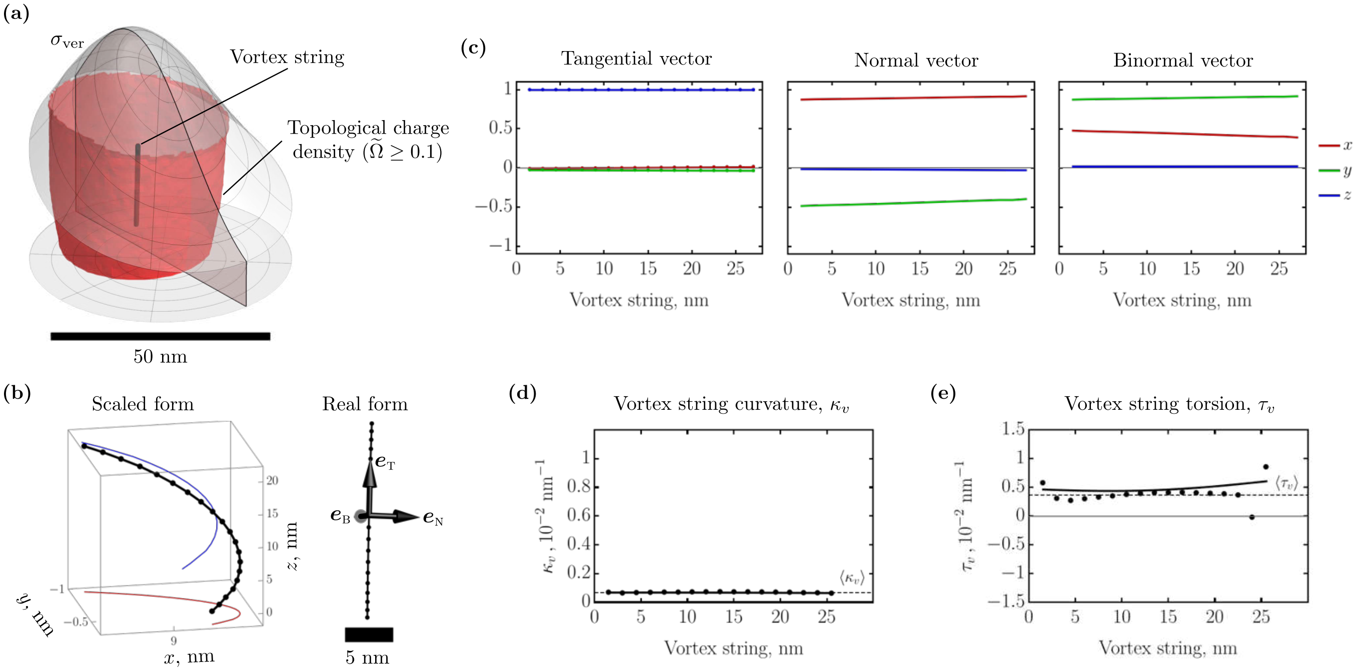

Supplementary Fig. 6. **Vortex state in a highly curved thin asymmetric nanodisk with $P = +1$ and $C = +1$ ($\widetilde{C} = +1$).** **(a)** Nanodisk geometry with $q = 10\,\text{nm}$, $t = 50\,\text{nm}$, $b = 20\,\text{nm}$ and $h = 5\,\text{nm}$ has almost straight vortex string pinned at the apex region of the Gaussian bump. **(b)** Left panel shows the vortex string scaled along $\hat{\boldsymbol{x}}$ and $\hat{\boldsymbol{y}}$ directions extracted from the center of mass of the topological charge distribution in the nanodisk, $\widetilde{\Omega}$. Red and blue lines indicate the $\hat{\boldsymbol{x}}\hat{\boldsymbol{y}}$ and $\hat{\boldsymbol{x}}\hat{\boldsymbol{z}}$ projections of the vortex string, respectively. Right panel shows the vortex string in 1:1:1 scale for all axes and local orthogonal frame of references formed by the tangential, $\boldsymbol{e}_{\rm T}$, normal, $\boldsymbol{e}_{\rm N}$, and binormal, $\boldsymbol{e}_{\rm B}$, vectors. **(c)** Components of the tangential, normal and binormal vectors along the vortex string. Symbols show on-site values for the tangential direction, while solid lines indicate the smoothed trend lines, which are used for the analysis of curvature, κ_v, and torsion, τ_v, of the vortex string. **(d)** κ_v and **(e)**τ_v distributions along the vortex string. Symbols indicate on-site values, solid lines represent distribution trends and dashed lines show mean values $\langle\kappa_v\rangle$ and $\langle\tau_v\rangle$.

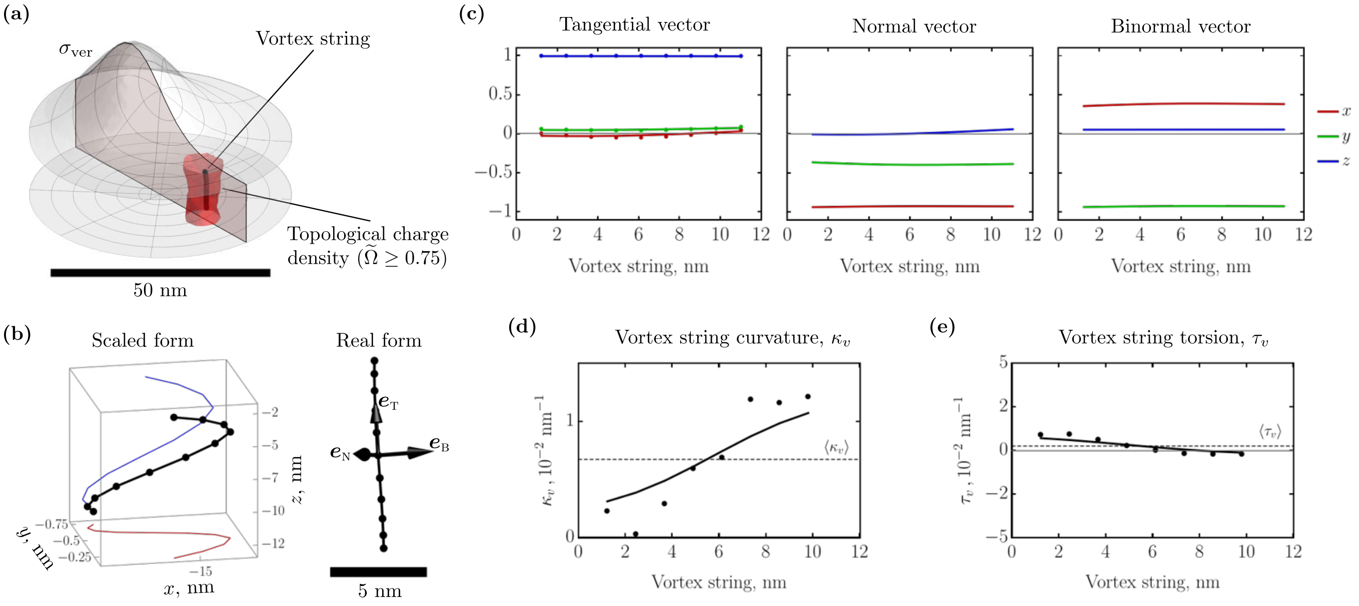

Supplementary Fig. 7. **Vortex state in thin asymmetric nanodisk with $P = +1$ and $C = +1$ ($\widetilde{C} = +1$). (a)** Nanodisk geometry with $q = 10\,\mathrm{nm}$, $t = 20\,\mathrm{nm}$, $b = 10\,\mathrm{nm}$ and $h = 15\,\mathrm{nm}$ has almost straight vortex string shifted to the bump tail along the mirror symmetry σ_{ver} plane. **(b)** Scaled (left panel) and real (right panel) representation of the vortex string extracted from the center of mass of the topological charge with the indicated projections and local orthogonal frame of references. **(c)** Components of the tangential, normal and binormal vectors along the vortex string shown in panel (b). Symbols show on-site values for the tangential direction and solid lines shows the smoothed trend lines, which are used for analysis. Distribution of **(d)** curvature and **(e)** torsion along the vortex string (symbols). Dashed lines show mean values $\langle\kappa_v\rangle$ and $\langle\tau_v\rangle$. Solid lines show the trend.

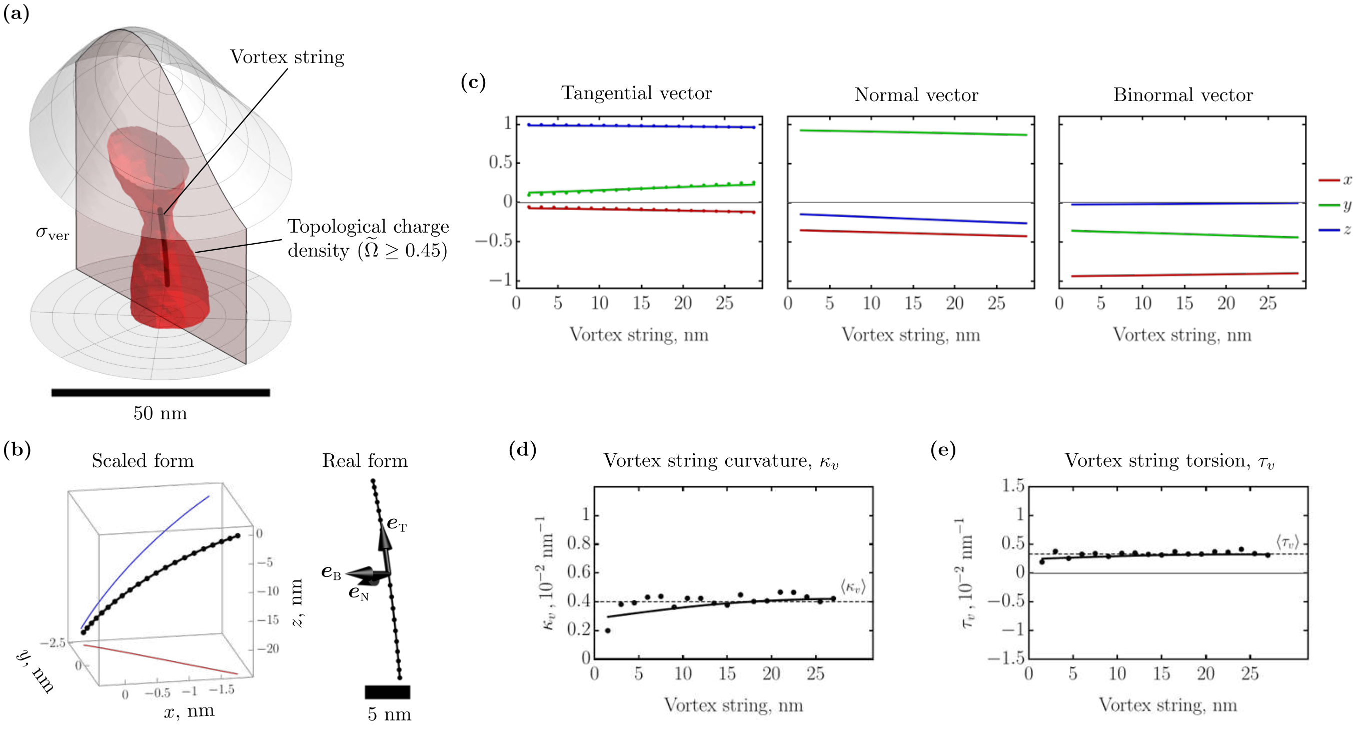

Supplementary Fig. 8. **Vortex state in thin asymmetric nanodisk with** $P = +1$ **and** $C = +1$ ($\widetilde{C} = +1$)**.** **(a)** Nanodisk geometry with $q = 10\,\text{nm}$, $t = 40\,\text{nm}$, $b = 20\,\text{nm}$ and $h = 30\,\text{nm}$ has almost straight vortex string shifted to the bump tail along the mirror symmetry σ_{ver} plane. **(b)** Scaled (left panel) and real (right panel) representations of the vortex string extracted from the center of mass of the topological charge with the indicated projections and local orthogonal frame of references. **(c)** Components of the tangential, normal and binormal vectors along the vortex string shown in panel (b). Symbols show on-site values for the tangential direction and solid lines shows the smoothed trend lines, which are used for analysis. Distribution of **(d)** curvature and **(e)** torsion along the vortex string (symbols). Dashed lines show mean values $\langle\kappa_v\rangle$ and $\langle\tau_v\rangle$. Solid lines show the trend.

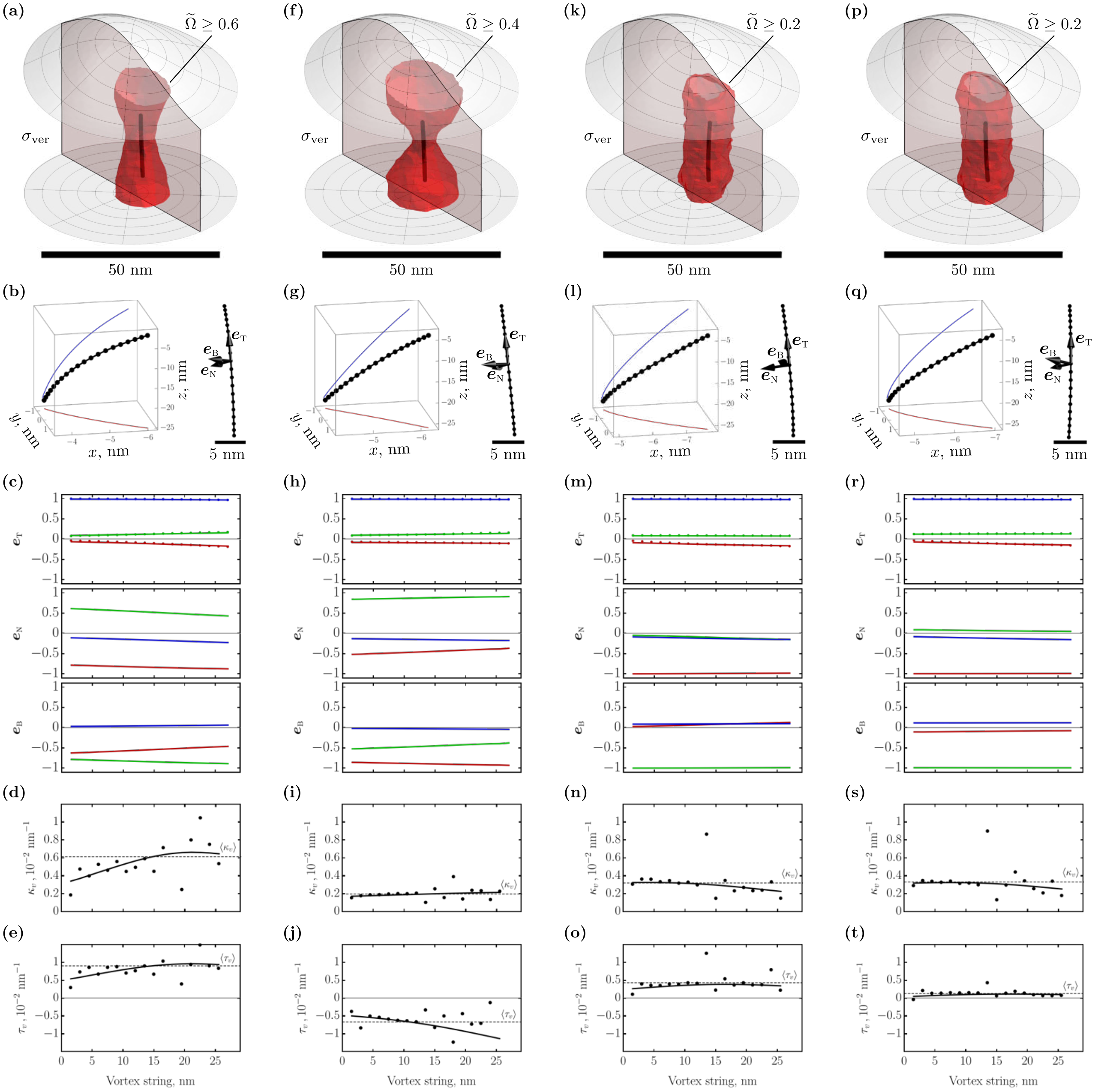

Supplementary Fig. 9. **Comparison of the vortex states with $P = +1$ and $C = +1$ in an asymmetric nanodisk with σ_{ver} symmetry ($q = 10$ nm, $t = 20$ nm, $b = 20$ nm and $h = 30$ nm) for different magnetic models. (a)** The resulting vortex string (black line) obtained from the full-scale micromagnetic simulations for ten different meshes and calculated from the distribution of $\widetilde{\Omega}$ by means of Eq. (S2). The red region corresponds to the area with $\widetilde{\Omega} \in [0.6, 1]$. **(b)** Scaled (left panel) and real (right panel) shapes of the vortex string extracted from the center of mass of the normalized topological charge $\widetilde{\Omega}$. In the left panel, red and blue lines correspond to the $\hat{\boldsymbol{x}}\hat{\boldsymbol{y}}$ and $\hat{\boldsymbol{x}}\hat{\boldsymbol{z}}$ plane projections. The right panel shows the directions of the tangential, $\boldsymbol{e}_{\text{T}}$, normal, $\boldsymbol{e}_{\text{N}}$, and binormal, $\boldsymbol{e}_{\text{B}}$, vectors. **(c)** Components of the tangential, normal and binormal vectors along the vortex string, which are calculated using the trend solid lines for the tangential vector. Symbols show on-site values for the tangential direction. Distributions of **(d)** curvature and **(e)** torsion along the vortex string (symbols). Dashed lines show mean values $\langle\kappa\rangle$ and $\langle\tau\rangle$. Solid line shows the trend. **(f–j)** Same for the for the model, in which the magnetization is pinned at all surface sites in the absence of magnetostatics. **(k–o)** Same for the model, in which the magnetization is pinned at all surface sites and the in-plane anisotropy with the hard axis of magnetization along $\hat{\boldsymbol{z}}$ (this model is referred to as model (i) in Supplementary Note 5). **(p–t)** Same for the model, in which magnetization is pinned at all surface sites and the in-surface anisotropy with the easy surface of magnetization gradually extruded from the flat bottom to the curved top surface of the sample (this model is referred to as model (ii) in Supplementary Note 5).

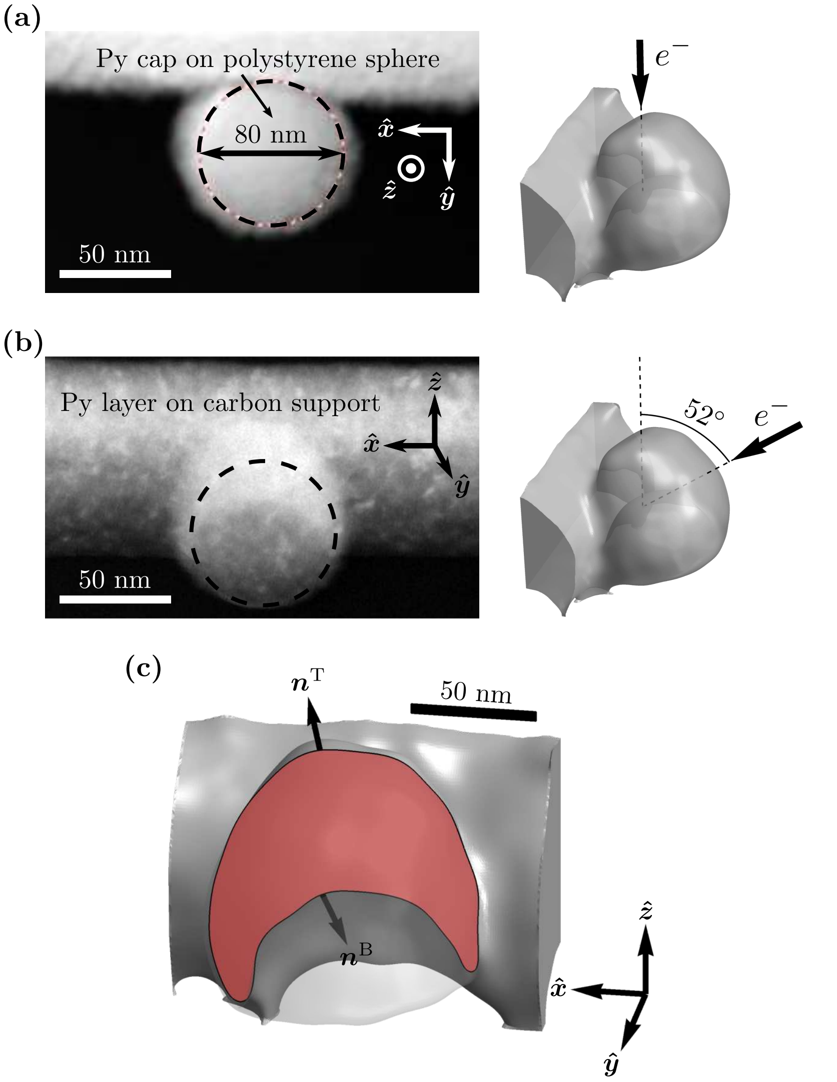

Supplementary Fig. 10. **Structural investigations of an asymmetric permalloy cap.** **(a)** Transmission electron microscopy (TEM) image of the permalloy nanocap formed on a polystyrene nanosphere with a diameter of 80 nm (dashed circle) being in contact with a supporting carbon frame. The right inset is a 3D schematics of the experimental geometry, which is shown as a top view image (0° altitude angle). **(b)** A side view TEM image taken at a 52° angle reveals the presence of inhomogeneous thickness distribution across the cap being 50 nm at the thickest part. **(c)** depicts a 3D cap volume obtained by means of bright-field TEM tomography. The cross-cut profile of the permalloy cap shows all features of the top and bottom surfaces with $\boldsymbol{n}^{\mathrm{T}}$ and $\boldsymbol{n}^{\mathrm{B}}$ being surface normal vectors to the top and bottom surfaces, respectively. The front part of the cap is shown semi-transparent.

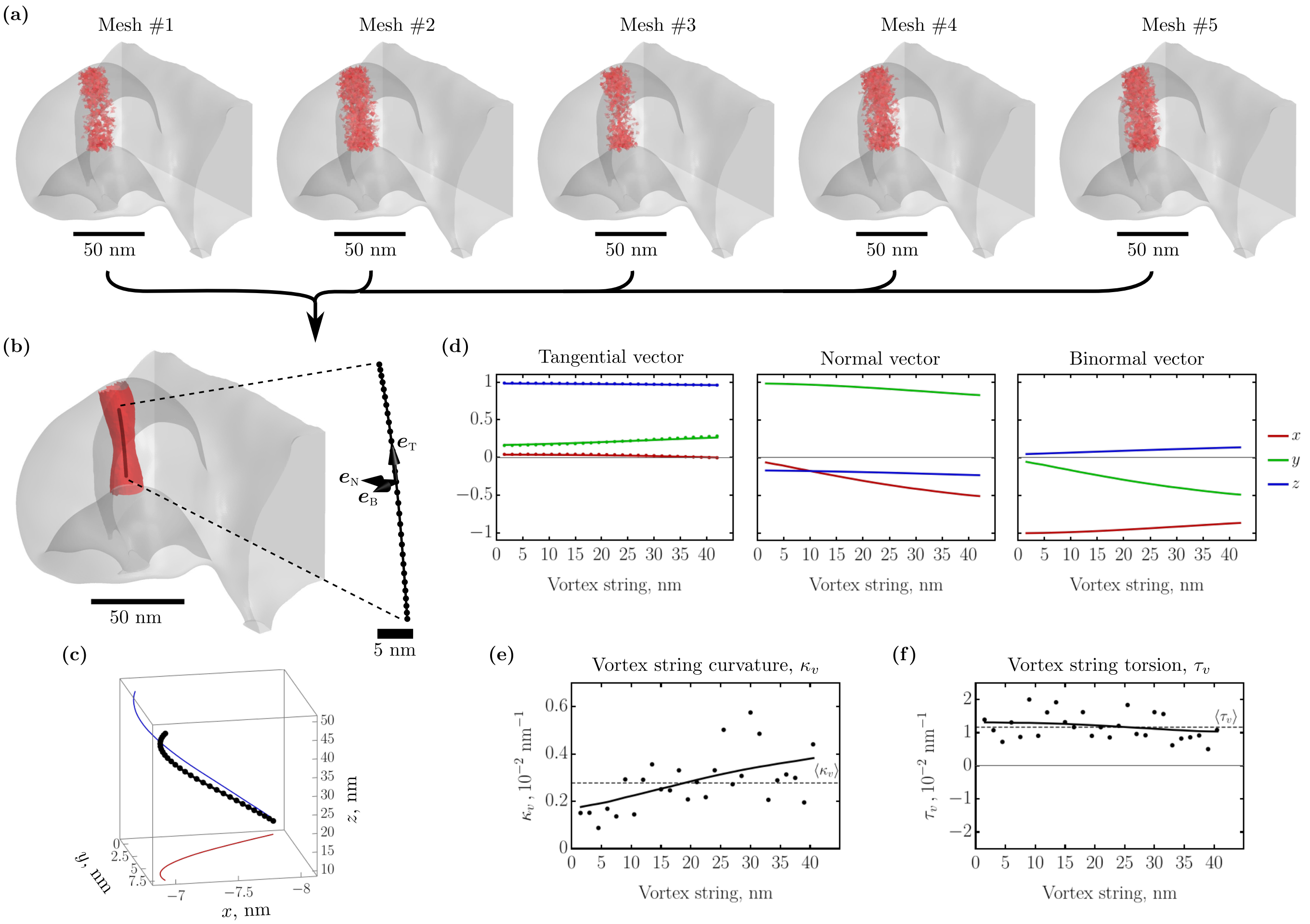

Supplementary Fig. 11. **Vortex state with $P = +1$ and $C = +1$ in the experimental geometry (simulations).** **(a)** The resulting distribution of tetrahedrons with $\widetilde{\Omega} \in [0.1, 1]$ (red) for different FEM meshes used in simulations. **(b)** Reconstructed vortex line (black tube) based on the data shown in panel (a) and the interpolated distribution for $\widetilde{\Omega} = 0.35$ for the Mesh #1. Right panel represents the real shape of the vortex string with the indicated tangential, $\boldsymbol{e}_{\mathrm{T}}$, normal, $\boldsymbol{e}_{\mathrm{N}}$, and binormal, $\boldsymbol{e}_{\mathrm{B}}$, vectors. **(c)** The scaled shape of the vortex string extracted from the calculation of $\boldsymbol{\gamma}$ in Eq. (S2). **(d)** Components of the tangential, normal and binormal vectors along the vortex string. Symbols show on-site values for the tangential direction and solid lines shows the smoothed trend line, which is used for analysis and calculation of normal and binormal vectors. Distribution of **(e)** curvature, κ_v, and **(f)** torsion, τ_v, along the vortex string (symbols). Dashed lines show mean values $\langle\kappa_v\rangle$ and $\langle\tau_v\rangle$. Solid lines show trends.

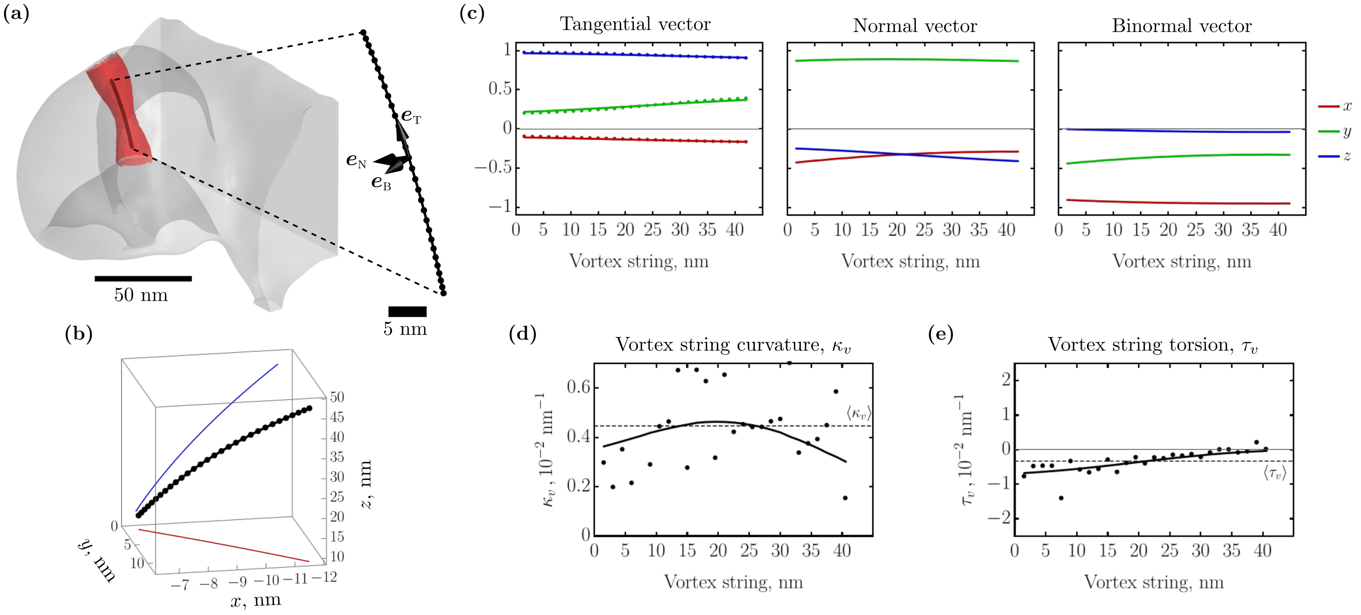

Supplementary Fig. 12. **Vortex state with $P = -1$ and $C = +1$ in the experimental geometry (simulations).** **(a)** Reconstructed vortex line (black tube) based on the data shown in panel (a) and the interpolated distribution for $\widetilde{\Omega} = 0.35$ for the Mesh #1. Right panel represents the real shape of the vortex string with the indicated tangential, $\boldsymbol{e}_\mathrm{T}$, normal, $\boldsymbol{e}_\mathrm{N}$, and binormal, $\boldsymbol{e}_\mathrm{B}$, vectors. **(b)** The scaled shape of the vortex string extracted from the calculation of $\widetilde{\Omega}$, see Eq. (S2). **(c)** Components of the tangential, normal and binormal vectors along the vortex string. Symbols show on-site values for the tangential direction and solid lines shows the smoothed trend line, which is used for analysis and calculation of normal and binormal vectors. Distribution of **(d)** curvature, κ_v, and **(e)** torsion, τ_v, along the vortex string (symbols). Dashed lines show mean values $\langle\kappa_v\rangle$ and $\langle\tau_v\rangle$. Solid lines show trends.

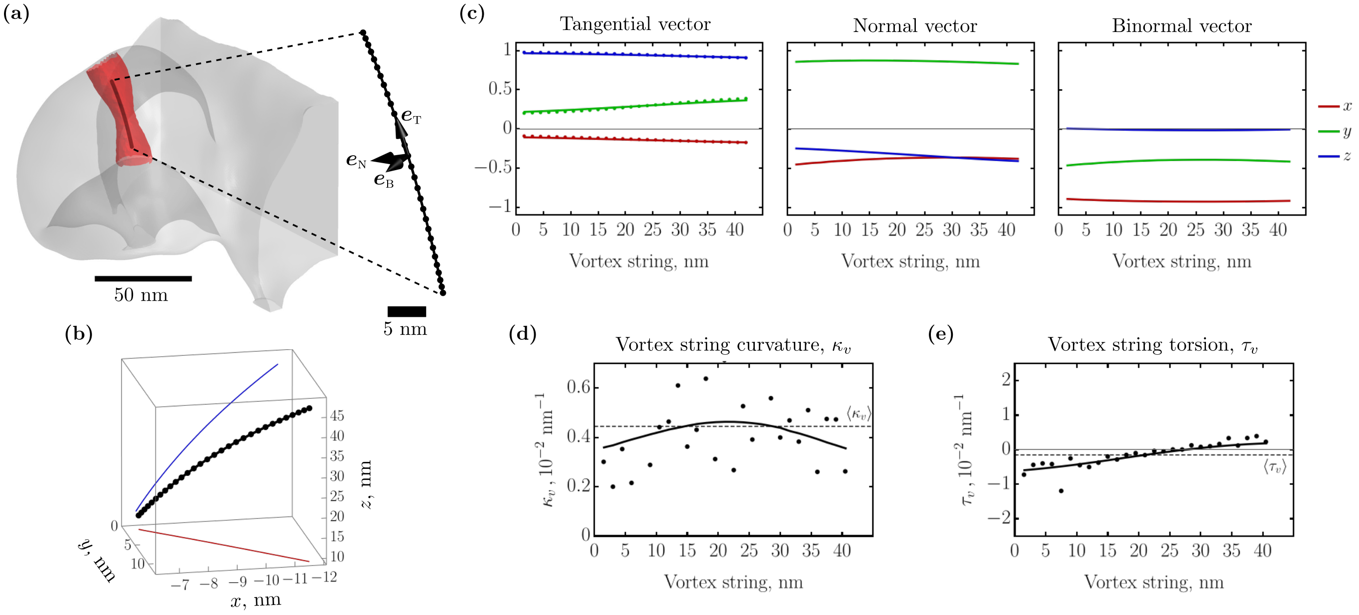

Supplementary Fig. 13. **Vortex state with $P = +1$ and $C = -1$ in the experimental geometry (simulations).** **(a)** Reconstructed vortex line (black tube) based on the data shown in panel (a) and the interpolated distribution for $\widetilde{\Omega} = 0.35$ for the Mesh #1. Right panel represents the real shape of the vortex string with the indicated tangential, $\boldsymbol{e}_{\mathrm{T}}$, normal, $\boldsymbol{e}_{\mathrm{N}}$, and binormal, $\boldsymbol{e}_{\mathrm{B}}$, vectors. **(b)** The scaled shape of the vortex string extracted from the calculation of $\widetilde{\Omega}$, see Eq. (S2). **(c)** Components of the tangential, normal and binormal vectors along the vortex string. Symbols show on-site values for the tangential direction and solid lines shows the smoothed trend line, which is used for analysis and calculation of normal and binormal vectors. Distribution of **(d)** curvature, κ_v, and **(e)** torsion, τ_v, along the vortex string (symbols). Dashed lines show mean values $\langle\kappa_v\rangle$ and $\langle\tau_v\rangle$. Solid lines show trends.

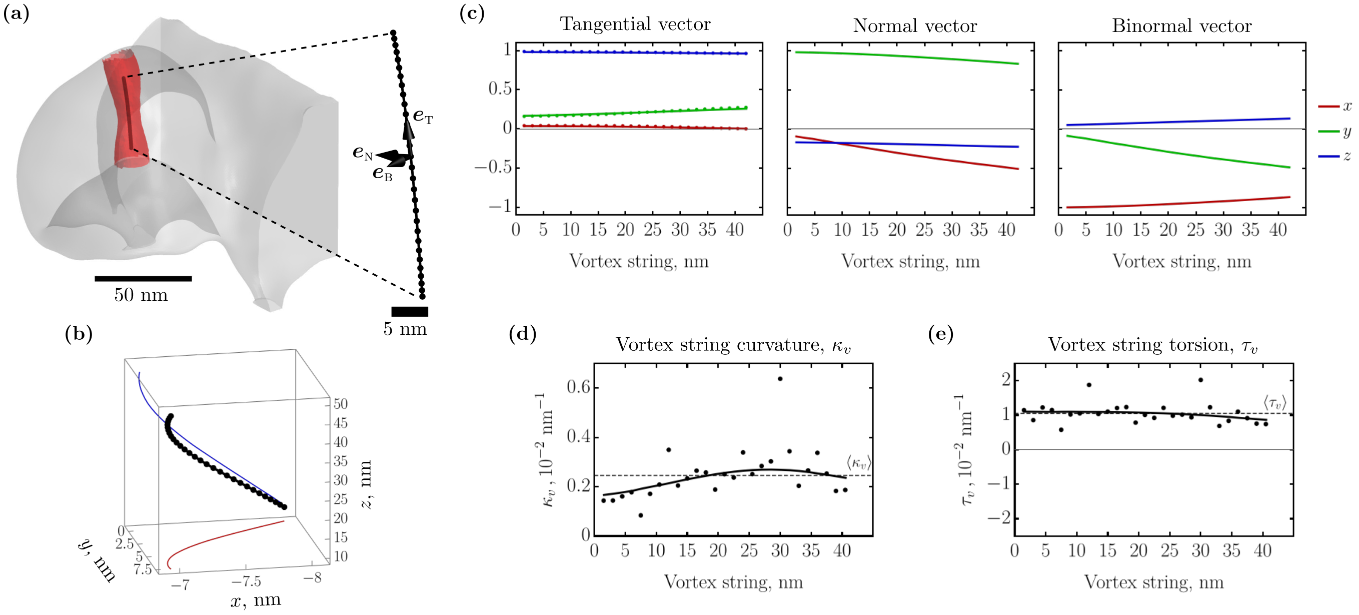

Supplementary Fig. 14. **Vortex state with $P = -1$ and $C = -1$ in the experimental geometry (simulations).** **(a)** Reconstructed vortex line (black tube) based on the data shown in panel (a) and the interpolated distribution for $\widetilde{\Omega} = 0.35$ for the Mesh #1. Right panel represents the real shape of the vortex string with the indicated tangential, $\boldsymbol{e}_{\mathrm{T}}$, normal, $\boldsymbol{e}_{\mathrm{N}}$, and binormal, $\boldsymbol{e}_{\mathrm{B}}$, vectors. **(b)** The scaled shape of the vortex string extracted from the calculation of $\widetilde{\Omega}$, see Eq. (S2). **(c)** Components of the tangential, normal and binormal vectors along the vortex string. Symbols show on-site values for the tangential direction and solid lines shows the smoothed trend line, which is used for analysis and calculation of normal and binormal vectors. Distribution of **(d)** curvature, κ_v, and **(e)** torsion, τ_v, along the vortex string (symbols). Dashed lines show mean values $\langle\kappa_v\rangle$ and $\langle\tau_v\rangle$. Solid lines show trends.

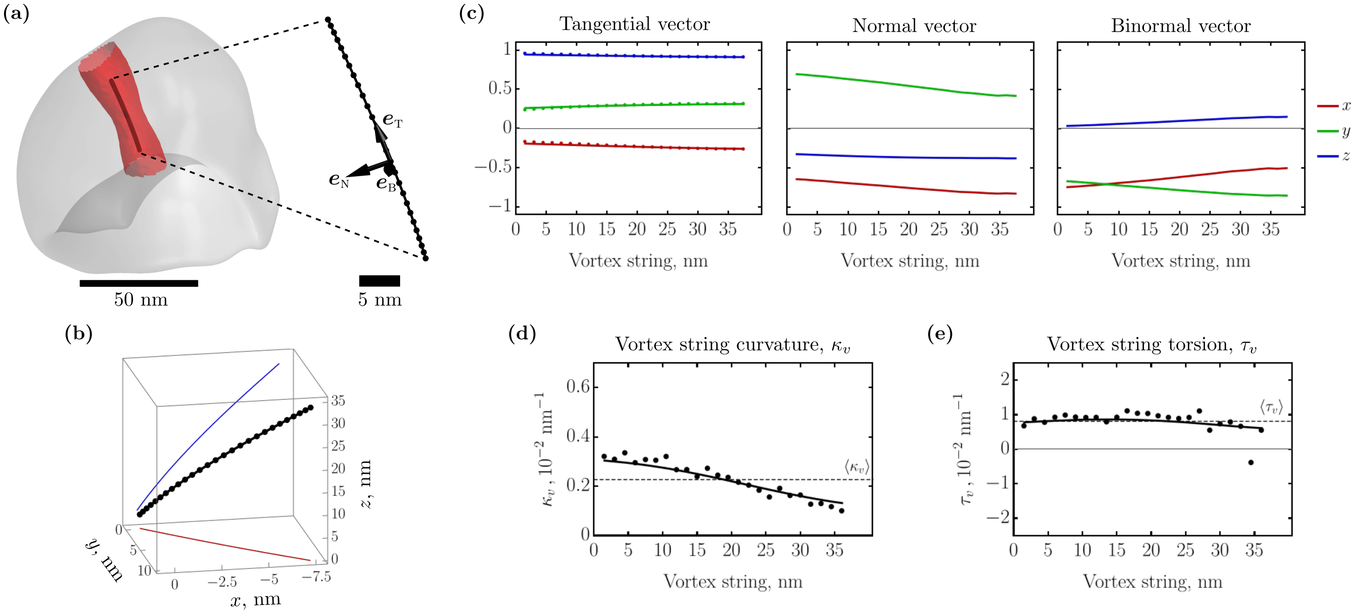

Supplementary Fig. 15. **Vortex state with $P = +1$ and $C = +1$ in the truncated experimental nanocap.** **(a)** Reconstructed vortex line (black tube) for the truncated nanocap and the interpolated distribution for $\widetilde{\Omega} = 0.35$. Right panel represents the real shape of the vortex string with the indicated tangential, $\boldsymbol{e}_\mathrm{T}$, normal, $\boldsymbol{e}_\mathrm{N}$, and binormal, $\boldsymbol{e}_\mathrm{B}$, vectors. **(b)** The scaled shape of the vortex string extracted from the calculation of $\widetilde{\Omega}$, see Eq. (S2). **(c)** Components of the tangential, normal and binormal vectors along the vortex string. Symbols show on-site values for the tangential direction and solid lines shows the smoothed trend line, which is used for analysis and calculation of normal and binormal vectors. Distribution of **(d)** curvature, κ_v, and **(e)** torsion, τ_v, along the vortex string (symbols). Dashed lines show mean values $\langle\kappa_v\rangle$ and $\langle\tau_v\rangle$. Solid lines show trends.

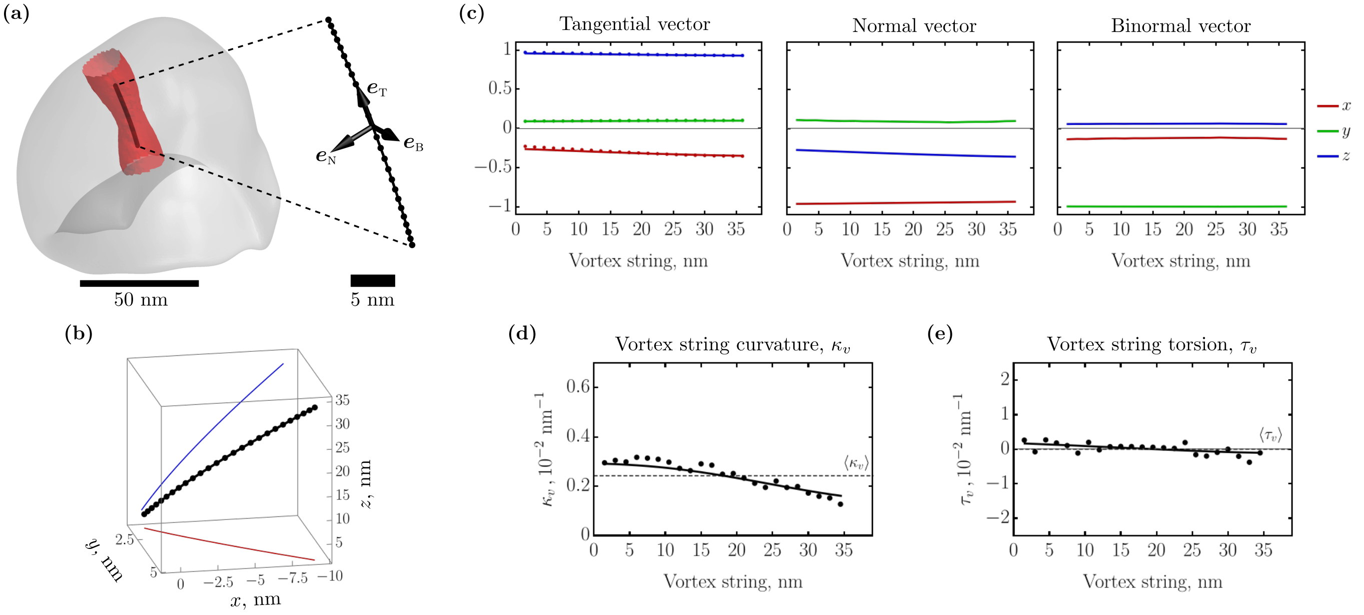

Supplementary Fig. 16. **Vortex state with $P = -1$ and $C = +1$ in the truncated experimental nanocap.** **(a)** Reconstructed vortex line (black tube) for the truncated nanocap and the interpolated distribution for $\widetilde{\Omega} = 0.35$. Right panel represents the real shape of the vortex string with the indicated tangential, $\boldsymbol{e}_\mathrm{T}$, normal, $\boldsymbol{e}_\mathrm{N}$, and binormal, $\boldsymbol{e}_\mathrm{B}$, vectors. **(b)** The scaled shape of the vortex string extracted from the calculation of $\widetilde{\Omega}$, see Eq. (S2). **(c)** Components of the tangential, normal and binormal vectors along the vortex string. Symbols show on-site values for the tangential direction and solid lines shows the smoothed trend line, which is used for analysis and calculation of normal and binormal vectors. Distribution of **(d)** curvature, κ_v, and **(e)** torsion, τ_v, along the vortex string (symbols). Dashed lines show mean values $\langle\kappa_v\rangle$ and $\langle\tau_v\rangle$. Solid lines show trends.

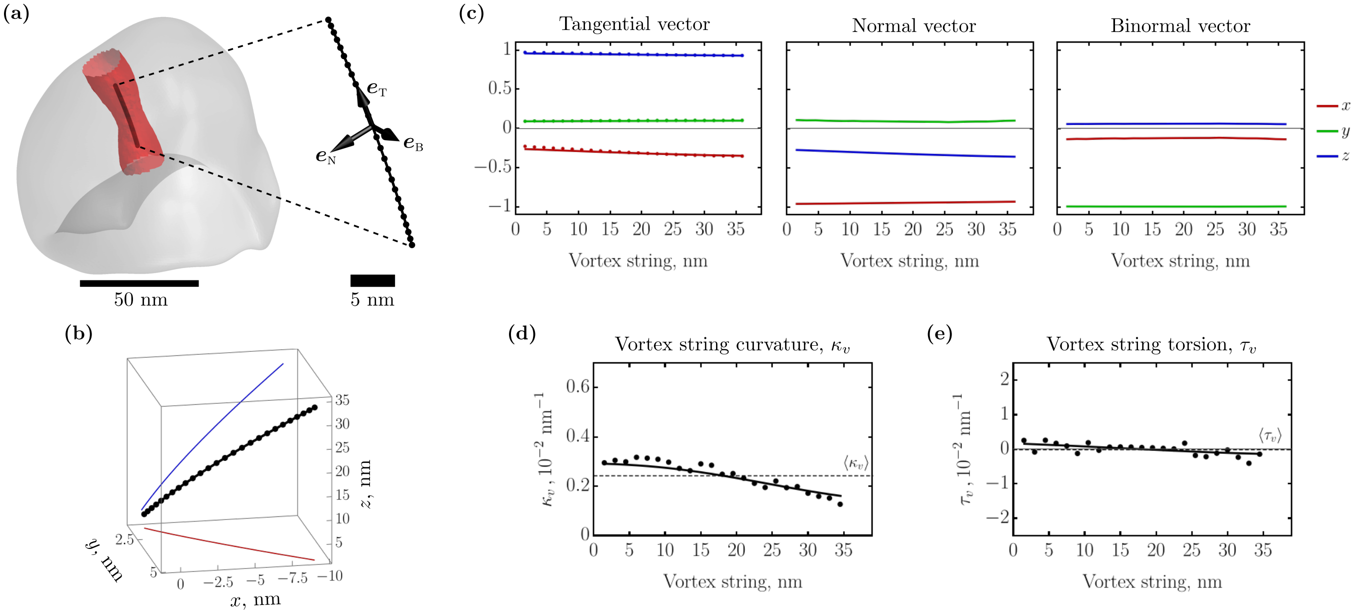

Supplementary Fig. 17. **Vortex state with** $P = +1$ **and** $C = -1$ **in the truncated experimental nanocap.** **(a)** Reconstructed vortex line (black tube) for the truncated nanocap and the interpolated distribution for $\widetilde{\Omega} = 0.35$. Right panel represents the real shape of the vortex string with the indicated tangential, $\boldsymbol{e}_{\mathrm{T}}$, normal, $\boldsymbol{e}_{\mathrm{N}}$, and binormal, $\boldsymbol{e}_{\mathrm{B}}$, vectors. **(b)** The scaled shape of the vortex string extracted from the calculation of $\widetilde{\Omega}$, see Eq. (S2). **(c)** Components of the tangential, normal and binormal vectors along the vortex string. Symbols show on-site values for the tangential direction and solid lines shows the smoothed trend line, which is used for analysis and calculation of normal and binormal vectors. Distribution of **(d)** curvature, κ_v, and **(e)** torsion, τ_v, along the vortex string (symbols). Dashed lines show mean values $\langle\kappa_v\rangle$ and $\langle\tau_v\rangle$. Solid lines show trends.

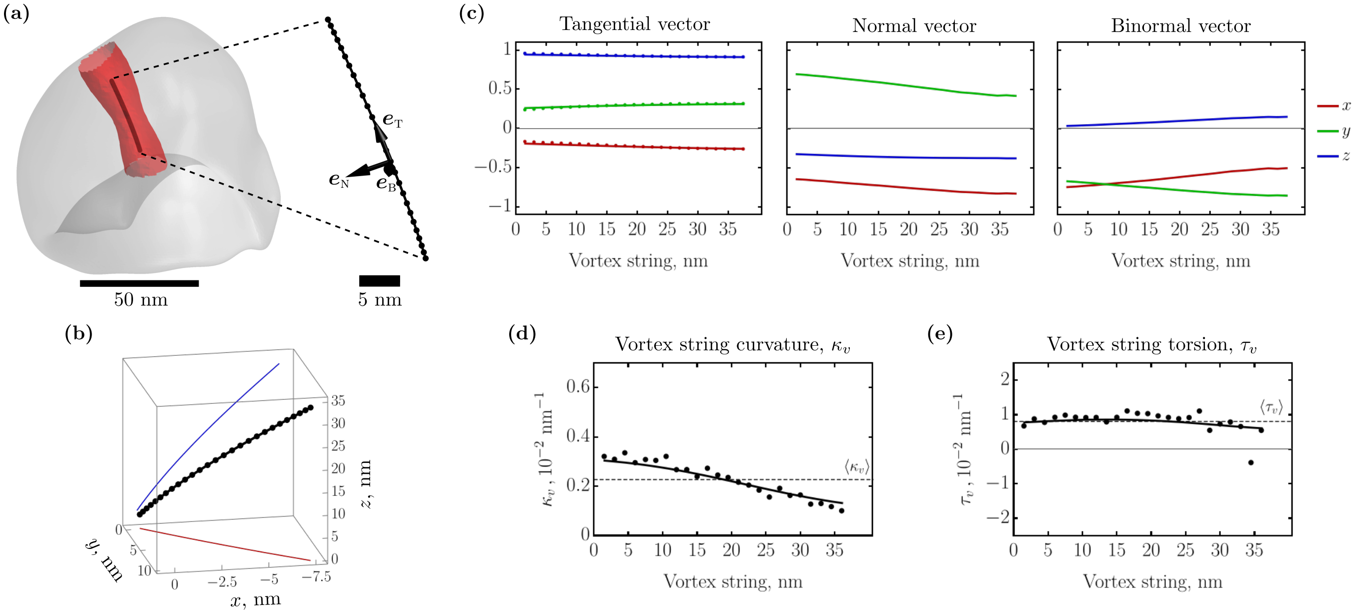

Supplementary Fig. 18. **Vortex state with $P = -1$ and $C = -1$ in the truncated experimental nanocap.** **(a)** Reconstructed vortex line (black tube) for the truncated nanocap and the interpolated distribution for $\widetilde{\Omega} = 0.35$. Right panel represents the real shape of the vortex string with the indicated tangential, $\boldsymbol{e}_{\text{T}}$, normal, $\boldsymbol{e}_{\text{N}}$, and binormal, $\boldsymbol{e}_{\text{B}}$, vectors. **(b)** The scaled shape of the vortex string extracted from the calculation of $\widetilde{\Omega}$, see Eq. (S2). **(c)** Components of the tangential, normal and binormal vectors along the vortex string. Symbols show on-site values for the tangential direction and solid lines shows the smoothed trend line, which is used for analysis and calculation of normal and binormal vectors. Distribution of **(d)** curvature, κ_v, and **(e)** torsion, τ_v, along the vortex string (symbols). Dashed lines show mean values $\langle\kappa_v\rangle$ and $\langle\tau_v\rangle$. Solid lines show trends.

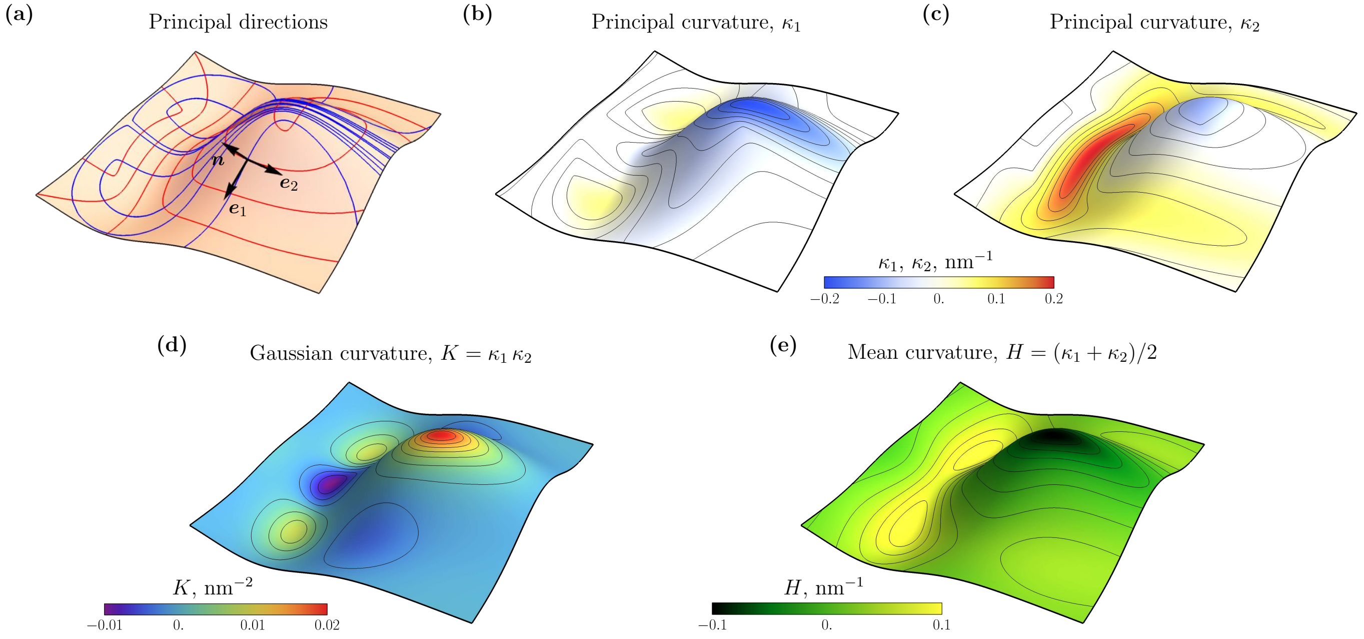

Supplementary Fig. 19. **Schematics of principal directions and principal curvatures on a curved surface.** **(a)** Local curvilinear frame of references constructed from the orthogonal vectors of principal directions $\boldsymbol{e}_{1,2}$ and surface normal $\boldsymbol{n}$. Blue and red curves show isolines along the same $\boldsymbol{e}_1$ and $\boldsymbol{e}_2$ principal directions, respectively. Panels **(b)** and **(c)** represent distributions of the principal curvatures κ_1 and κ_2 that correspond to the local maximal and minimal values of curvature, respectively. Color bar shows the local value of the principal curvatures $\kappa_{1,2}$. Panels **(d)** and **(e)** show distributions of the Gaussian curvature $K = \kappa_1\,\kappa_2$ and mean curvature $H = (\kappa_1 + \kappa_2)/2$, respectively.

Supplementary Tables

Supplementary Table I. **Comparison of the vortex string parameters for different models of the nanodisk with σ_{ver} symmetry.** Sample parameters are $t = 20\,\text{nm}$, $b = 20\,\text{nm}$ and $h = 30\,\text{nm}$. Here, "–" means absence of the interaction for the given simulation, "uniform" and "varying" labels for the Anisotropy column correspond to the models (i) and (ii) described in Supplementary Note 5.

Exchange	Magnetostatics	Anisotropy	$\boldsymbol{M}$ pinning	$\widetilde{C}$	$\langle\kappa_v\rangle$, μm^{-1}	$\langle\tau_v\rangle$, μm^{-1}	$\langle R_v\rangle$, ℓ	$\langle P_v\rangle$, ℓ	ψ
✓	✓	–	–	+1	6.2	9.0	9.7	89.5	-51°
✓	–	–	✓		2.0	-6.6	7.9	164.3	-53°
✓	–	uniform	✓		3.2	4.3	20.8	176.4	-24°
✓	–	varying	✓		3.3	1.3	48.7	123.0	-51°
✓	✓	–	–	-1	6.6	-5.2	17.7	87.4	$+59^\circ$
✓	–	–	✓		2.0	-5.4	11.6	192.5	$+41^\circ$
✓	–	uniform	✓		3.8	5.4	16.5	146.5	$+23$
✓	–	varying	✓		3.4	0.8	52.3	74.8	$+39$

Supplementary Table II. **Comparison of the vortex string parameters for magnetochiralities $\widetilde{C}$ of the asymmetric experimental nanocap.**

$\widetilde{C}$	P	C	$\langle\kappa_v\rangle$, $\mu\mathrm{m}^{-1}$	$\langle\tau_v\rangle$, $\mu\mathrm{m}^{-1}$	$\langle R_v\rangle$, ℓ	$\langle P_v\rangle$, ℓ
$+1$	$+1$	$+1$	2.7	11.7	3.6	95.5
	-1	-1	2.5	10.6	3.9	106.0
-1	$+1$	-1	4.4	-1.4	38.4	76.6
	-1	$+1$	4.5	-3.2	27.8	125.4

Supplementary Table III. **Comparison of the vortex string parameters for magnetochiralities $\widetilde{C}$ of the truncated experimental nanocap.**

$\widetilde{C}$	P	C	$\langle\kappa_v\rangle$, μm^{-1}	$\langle\tau_v\rangle$, μm^{-1}	$\langle R_v\rangle$, ℓ	$\langle P_v\rangle$, ℓ
$+1$	$+1$	$+1$	2.3	8.2	6.0	134.4
	-1	-1	2.3	8.2	6.0	134.8
-1	$+1$	-1	2.4	-0.05	77.4	9.4
	-1	$+1$	2.4	0.12	77.2	23.5

References

[1] J. Raabe, R. Pulwey, R. Sattler, T. Schweiboeck, J. Zweck, and D. Weiss, Magnetization pattern of ferromagnetic nanodisks, Journal of Applied Physics **88**, 4437 (2000).

[2] T. Shinjo, T. Okuno, R. Hassdorf, K. Shigeto, and T. Ono, Magnetic vortex core observation in circular dots of permalloy, Science **289**, 930 (2000).

[3] M. Schneider, H. Hoffmann, and J. Zweck, Lorentz microscopy of circular ferromagnetic permalloy nanodisks, Applied Physics Letters **77**, 2909 (2000).

[4] R. Hertel and C. M. Schneider, Exchange explosions: Magnetization dynamics during vortex-antivortex annihilation, Physical Review Letters **97**, 177202 (2006).

[5] N. Papanicolaou and T. N. Tomaras, Dynamics of magnetic vortices, Nuclear Physics B **360**, 425 (1991).

[6] N. Papanicolaou, Dynamics of magnetic vortex rings, in *Singularities in fluids, plasmas, and optics*, Vol. 404 (Springer Netherlands, 1993).

[7] N. R. Cooper, Propagating magnetic vortex rings in ferromagnets, Physical Review Letters **82**, 1554 (1999).

[8] C. Donnelly, K. L. Metlov, V. Scagnoli, M. Guizar-Sicairos, M. Holler, N. S. Bingham, J. Raabe, L. J. Heyderman, N. R. Cooper, and S. Gliga, Experimental observation of vortex rings in a bulk magnet, Nature Physics **17**, 316 (2020).

[9] V. P. Kravchuk, D. D. Sheka, U. K. Rößler, J. van den Brink, and Y. Gaididei, Spin eigenmodes of magnetic skyrmions and the problem of the effective skyrmion mass, Physical Review B **97**, 064403 (2018).

[10] V. P. Kravchuk, U. K. Rößler, J. van den Brink, and M. Garst, Solitary wave excitations of skyrmion strings in chiral magnets, Physical Review B **102**, 10.1103/physrevb.102.220408 (2020).

[11] D. Carroll, E. Hankins, E. Kose, and I. Sterling, A survey of the differential geometry of discrete curves, **36**, 28.

[12] A. Hubert and R. Schäfer, *Magnetic domains: The analysis of magnetic microstructures* (Springer Berlin Heidelberg, Berlin, 2009).

[13] H. K. Moffatt, en*Magnetic field generation in electrically conducting fluids*, Cambridge monographs on mechanics and applied mathematics (Cambridge University Press, Cambridge, England, 1983).

[14] S. Molokov, R. Moreau, and K. Moffatt, *Magnetohydrodynamics: Historical Evolution and Trends*, 1st ed., Fluid Mechanics And Its Applications 80 (Springer Netherlands, 2007).

[15] G. Volovik, *The universe in a Helium droplet* (Oxford University Press, Oxford, 2003).

[16] Y. Gaididei, D. D. Sheka, and F. G. Mertens, Controllable switching of vortex chirality in magnetic nanodisks by a field pulse, Applied Physics Letters **92**, 012503 (2008).

[17] S.-B. Choe, Y. Acremann, A. Scholl, A. Bauer, A. Doran, J. Stöhr, and H. A. Padmore, Vortex core-driven magnetization dynamics, Science **304**, 420 (2004).

[18] M.-Y. Im, P. Fischer, K. Yamada, T. Sato, K. S., Y. Nakatani, and T. Ono, Symmetry breaking in the formation of magnetic vortex states in a permalloy nanodisk, Nature Communications **3**, 983 (2012).

[19] H. A. M. van den Berg, Self-consistent domain theory in soft ferromagnetic media. i. solenoidal distributions in elliptical thin-film elements, Journal of Applied Physics **57**, 2168 (1985).

[20] H. A. M. van den Berg, Self-consistent domain theory in soft-ferromagnetic media. ii. basic domain structures in thin-film objects, Journal of Applied Physics **60**, 1104 (1986).

[21] J. R. Ferraro and J. S. Ziomek, *Introductory Group Theory* (Springer US, Boston, MA, 1969).

[22] T. Moriya, Anisotropic superexchange interaction and weak ferromagnetism, Physical Review **120**, 91 (1960).

[23] I. E. Dzyaloshinskii, Theory of helicoidal structures in antiferromagnets. i. nonmetals, Sov. Phys. JETP **19**, 960 (1964).

[24] A. B. Butenko, A. A. Leonov, A. N. Bogdanov, and U. K. Rößler, Theory of vortex states in magnetic nanodisks with induced Dzyaloshinskii-Moriya interactions, Physical Review B **80**, 134410 (2009).

[25] mumax3, developed by DyNaMat group of Prof. Van Waeyenberge at Ghent University.

[26] A. Vansteenkiste, J. Leliaert, M. Dvornik, M. Helsen, F. Garcia-Sanchez, and B. Van Waeyenberge, The design and verification of MuMax3, AIP Advances **4**, 107133 (2014).

[27] O. A. Tretiakov, M. Morini, S. Vasylkevych, and V. Slastikov, Engineering curvature-induced anisotropy in thin ferromagnetic films, Physical Review Letters **119**, 077203 (2017).

[28] MAGPAR finite element micromagnetics package, developed by Werner Scholz.

[29] W. Scholz, K. Y. Guslienko, V. Novosad, D. Suess, T. Schrefl, R. W. Chantrell, and J. Fidler, Transition from single-domain to vortex state in soft magnetic cylindrical nanodots, Journal of Magnetism and Magnetic Materials **266**, 155 (2003).

[30] G. Taubin, Estimating the tensor of curvature of a surface from a polyhedral approximation, in *Proceedings of IEEE International Conference on Computer Vision* (IEEE Comput. Soc. Press, 1995) 902-907.
