# Supplementary material for: Chirality coupling in topological magnetic textures with multiple magnetochiral parameters
Source: Nat Commun. 2023 Mar 17;14:1491. doi: 10.1038/s41467-023-37081-z (PMC10023801; doi:10.1038/s41467-023-37081-z)
Supplement: Supplementary file 4 — Description of Additional Supplementary Files [file 41467_2023_37081_MOESM4_ESM.pdf]

Title: Supplementary Movie 1

Description: The movie shows a 3D volume rendering of a permalloy cap (shown in the front at the starting frame) located on a permalloy-covered carbon support (shown in the back at the starting frame). The color-coded volume rendering visualizes the reconstructed density, which is proportional to the attenuation coefficient caused by the scattering absorption of the objective lens aperture in the transmission electron microscope (TEM). The voxel size is $0.5\times0.5\times0.5$~nm^3. The animation starts with a 360° rotation around the vertical axis, followed by a second 360° rotation with additional visualization of the segmented surface. The movie was created using the Avizo software package (ThermoFisher Scientific Company).
